# Spherical-Symmetry and Spin Effects on the Uncertainty Measures of Multidimensional Quantum Systems with Central Potentials

**DOI:** 10.3390/e23050607

**Published:** 2021-05-14

**Authors:** Jesús S. Dehesa

**Affiliations:** 1 Departamento de Física Atómica, Molecular y Nuclear, Universidad de Granada, 18071 Granada, Spain; dehesa@ugr.es; 2 Instituto Carlos I de Física Teórica y Computacional, Universidad de Granada, 18071 Granada, Spain

**Keywords:** central potentials, uncertainty relations, integral inequalities, Heisenberg-like uncertainty measures, entropy-like measures, Fisher information, Shannon entropy, Rényi entropies

## Abstract

The spreading of the stationary states of the multidimensional single-particle systems with a central potential is quantified by means of Heisenberg-like measures (radial and logarithmic expectation values) and entropy-like quantities (Fisher, Shannon, Rényi) of position and momentum probability densities. Since the potential is assumed to be analytically unknown, these dispersion and information-theoretical measures are given by means of inequality-type relations which are explicitly shown to depend on dimensionality and state’s angular hyperquantum numbers. The spherical-symmetry and spin effects on these spreading properties are obtained by use of various integral inequalities (Daubechies–Thakkar, Lieb–Thirring, Redheffer–Weyl, ...) and a variational approach based on the extremization of entropy-like measures. Emphasis is placed on the uncertainty relations, upon which the essential reason of the probabilistic theory of quantum systems relies.

## 1. Introduction

The central field approximation has been successfully applied to investigate the natural systems not only in the three-dimensional world, but also in multidimensional physics. The central field model of the atom and the Pauli exclusion principle are the fundamental building blocks of the construction principle of Mendeleev’s atomic periodic table [1,2,3,4,5,6]. Multidimensional central potentials with a specific analytical form (e.g., power-law, oscillator, Coulomb, van der Waals, Morse, Pöschl–Teller, Hulthen, Woods-Saxon, convex, Yukawa, ...) have been used to interpret a great deal of physical phenomena and chemical processes [7,8,9,10,11,12,13,14,15]. They have been applied to study the behaviour of nanotechnological systems (e.g., quantum dots and wires) and to explain the experiments of dilute systems in magnetic traps at extremely low temperatures [16,17,18], which has allowed for a fast development of a density-functional theory of independent particles in multidimensional central potentials [11,19]. The *D*-dimensional scaling method of Dudley R. Herschbach et al. [6], whose starting point is the high dimensionality limit, is able to describe the physical and chemical properties of finite many-electron systems with an accuracy similar to the self-consistent Hartree–Fock approaches; keep in mind that in both Herschbach and Hartree–Fock methods the average potential which is ultimately diagonalized is spherically symmetric. Moreover, it is commonly believed at present that the idea of higher dimensionalities is the best basis to explain the unification of all forces of physics. Thus, a wealth of physical insight into standard (three-dimensional) systems is being obtained within a framework of higher dimensions in many areas ranging from the theory of atoms and molecules [6] to cosmology (see, e.g., [10]).

In this work we analize the knowledge of the spreading measures of the position and momentum probability densities of a *D*-dimensional central potential, which control the position-momentum uncertainty of the *D*-dimensional quantum systems moving under the action of a central potential. Since the analytical form of the central potential is assumed not to be known, most of the results of this work are given necessarily by means of inequality-type relations. Now, the stationary wavefunctions of such a potential factorize into an unknown radial part and an angular part given by the renowned hyperspherical harmonics $Y_{l,\{\mu \}}\left(\Omega_{D-1}\right)$ of degree *l* on the unit sphere $S_{D-1}$ [20,21], which is characterized by the D−1 angular hyperquantum numbers $\left(l\equiv \mu_{1},\mu_{2},\ldots ,\mu_{D-1}\right)$ with the integer values l=0,1,2,…, and $l\geq \mu_{2}\geq \ldots \geq \left|\mu_{D-1}\right|\geq 0$ (see next section for further details). Then, the spreading/uncertainty measures will be expressed by means of inequality-type relations which depend on these state’s angular hyperquantum numbers.

Here, we consider the following complementary spreading/uncertainty quantifiers in both position and momentum spaces: the Heisenberg-like measures (variance, radial expectation values), the logarithmic measures and the information-theoretical measures of Fisher [22,23,24], Shannon [25,26] and Rényi [27,28,29] types. The Heisenberg-like measures are the most popular, historically speaking, because of their conceptual simplicity and their usefulness for the quantum systems with position and momentum densities of unimodal type (esp. Gaussian and quasi-Gaussian). They quantify the separation of the region(s) of the probability concentration with respect to a specific point of the system’s domain (usually, the origin or the mean value); so, they are misleading and not adequate quantifiers for the quantum uncertainty of numerous physical systems with heavy-tailed and oscillating, multimodal densities [30,31,32,33]. To avoid such drawbacks and since the quantum probability densities of the potential’s bound states are strongly oscillating, except for a few ones such as e.g., the *S* states, the entropy-like measures are much more natural and appropriate to quantify the position-momentum uncertainty of the system. In addition these measures (i) characterize numerous fundamental and measurable quantities of physical systems [34], (ii) are cornerstones of two alternative formulations to classical thermodynamics [35,36], and (iii) are basic variables of the classical and quantum information theory and quantum technologies [37,38] since they quantify the classical and quantum information contents as well as the states’ quantum entanglement.

The Rényi entropies $R_{q}\left[\rho \right]$ provide a family of entropic measures of the density ρ, depending on a real parameter *q*, which includes the Shannon entropy S[ρ] as a limiting case. These entropic measures quantify many spreading facets of the quantum probability density ρ(r) all over the hyperspace, which encompass the intrinsic disorder and the geometrical profile of the quantum system. As measures of disorder/uncertainty, the Rényi entropies (which have very important physico-mathematical properties *per se* [31,39,40,41,42,43,44,45]) allow for a much wider quantitative range of applicability than Heisenberg’s measures. This permits, for example, an entropic formulation [46,47] for the uncertainty principle of quantum physics which is stronger [42,48] than the variance-based Heisenberg mathematical formulation [49] and its generalizations, the radial expectation values [50]. Additionally, a great deal of practical purposes for the Rényi entropies and their associated uncertainty relations abound in numerous scientific fields [31,42,43,47], ranging from quantum entanglement [51], Brown processes and pattern formation [52,53], chaotic systems and fractals [39,54,55] to ergodicity [56], quantum phase transition [57], squeezing of quantum fluctuation [58], Bose-Einstein condensates [59,60,61] and quantum-classical correspondence [62], among many other applications.

The translationally invariant Fisher information F[ρ] of the density ρ is a gradient functional of the density, so that it has a property of locality because of its sensitivity to local rearrangements/fluctuations of the density. This is opposite to the previous entropy-like measures which have a global character because they are power-like (Rényi) or logarithmic (Shannon) functionals of the density. The Fisher information (and its parameter-dependent form) [22,23,24] is closely related to the kinetic and Weizsäcker energies [63,64,65] of the system. This has allowed it for multiple and diverse scientific, technological and finantial applications [24], ranging from atomic and molecular physics [66,67,68], dilute gases [69], chemical processes [70,71] to quantum information and quantum technologies [72,73,74,75,76,77,78].

The structure of the paper is as follows. In Section 2 we examine the knowledge of the stationary wave functions (and the associated quantum densities) of the Schrödinger equation for arbitrary multidimensional central (spherically-symmetric) potentials in both position and momentum spaces. In Section 3 we analyze the multidimensional spreading of a single-particle system subject to a general *D*-dimensional central potential by means of the dispersion measures of position and momentum types. The associated Heisenberg-like and logarithmic uncertainty relations are also examined. In Section 4 we discuss the main properties of the information-theoretical measures (Fisher information [24] and the Shannon [25] and Rényi [27,28] entropies) of the multidimensional quantum states of arbitrary central potentials, which are necessarily of inequality type. Emphasis is made on the associated entropic uncertainty relations. In Section 5 we investigate the combined balance of the space and spin dimensionality effects on the Heisenberg-like uncertainty relation of general fermionic systems, and the space, spin and spherical effects on the Fisher-information-based uncertainty relation of fermionic systems with arbitrary central potentials. This is done by use of integral inequalities of Daubechies–Thakkar and Lieb–Thirring type and variational methods based on the extremization of the entropy-like measures. Finally, in Section 6 some conclusions and open problems are given.

## 2. The Multidimensional Problem for Central Potentials

In this section we briefly describe in hyperspherical coordinates the wavefunctions and the associated probability densities for the discrete stationary states of a non-relativistic *D*-dimensional (D⩾2) single-particle system subject to a spherically symmetric potential $V_{D}\left(r\right)$, where r=|r|, in both position and momentum spaces. Later on, we briefly describe the associated probability densities for the stationary quantum states of the system. Atomic units (i.e., $\hbar =m_{e}=e=1$) are used throughout the paper.

### 2.1. The Wavefunctions

The stationary states of this system are characterized by the wavefunctions given as

(1) $$\psi \left(r,t\right)=\Psi \left(r\right)\;e^{-iEt},$$

where (E,Ψ) are the time-independent solutions of the Schrödinger equation

(2) $$\left(-\frac{1}{2}{\vec{\nabla }}_{D}^{2}+V_{D}\left(r\right)\right)\Psi \left(r\right)=E\;\Psi \left(r\right),$$

where the position vector $r=\left(x_{1},\ldots ,x_{D}\right)=\left(r,\theta_{1},\theta_{2},\ldots ,\theta_{D-1}\right)\equiv \left(r,\Omega_{D-1}\right)$ in Cartesian and hyperspherical units, respectively, so that $r\equiv \left|r\right|=\sqrt{\sum_{i=1}^{D}x_{i}^{2}}\in \left[0,\;+\infty \right)$ and $x_{i}=r\left(\prod_{k=1}^{i-1}\sin\theta_{k}\right)\cos\theta_{i}$ for 1≤i≤D and with $\theta_{i}\in \left[0,\pi \right),i<D-1$, $\theta_{D-1}\equiv \varphi \in \left[0,2\pi \right)$. Moreover, the symbol ${\vec{\nabla }}_{D}$ denotes the *D*-dimensional gradient operator

(3) $${\vec{\nabla }}_{D}=\frac{\partial }{\partial r}\hat{r}+\frac{1}{r}\sum_{i=1}^{D-2}\frac{\partial }{\partial \theta_{i}}\hat{\theta_{i}}+\frac{1}{r\prod_{i=1}^{D-2}\sin\theta_{i}}\frac{\partial }{\partial \varphi }\hat{\varphi },$$

and its square is a generalized Laplacian operator [20,79,80] which can be expressed as

(4) $${\vec{\nabla }}_{D}^{2}=\frac{d^{2}}{dr^{2}}+\frac{D-1}{r}\frac{d}{dr}-\frac{\Lambda_{D-1}^{2}}{r^{2}}$$

where $\Lambda^{2}$ is a partial differential (non-radial) operator on the unit sphere $S_{D-1}$ (see e.g., [81,82]) given by

(5) $$\Lambda_{D-1}^{2}=-\sum_{i=1}^{D-1}\frac{{\left(\sin\theta_{i}\right)}^{i+1-D}}{{\left(\prod_{i=j-1}^{i-1}\sin\theta_{j}\right)}^{2}}\frac{\partial }{\partial \theta_{i}}\left[{\left(\sin\theta_{i}\right)}^{D-1}\frac{\partial }{\partial \theta_{i}}\right]$$

This operator, which denotes the *D*-dimensional generalization of the square of the angular momentum, fulfills the eigenvalue equation

(6) $$\Lambda_{D-1}^{2}\;Y_{l,\left\{\mu \right\}}\left(\Omega_{D-1}\right)=l\left(l+D-2\right)\;Y_{l,\left\{\mu \right\}}\left(\Omega_{D-1}\right),$$

where the D−1 angular hyperquantum numbers $\left(l,\left\{\mu \right\}\right)\equiv \left(\mu_{1},\mu_{2},\ldots ,\mu_{D-1}\right)$ have the integer values l=0,1,2,…, and $l\geq \mu_{2}\geq \ldots \geq |\mu_{D-1}|\equiv |m|\geq 0$; note that for D=2 we only have one quantum number l∈Z. The symbol $Y_{l,\{\mu \}}\left(\Omega_{D-1}\right)$ denotes the known hyperspherical harmonics of degree *l* on the unit sphere $S_{D-1}$ which can be expressed [20,21,83] as

(7) $$Y_{l,\left\{\mu \right\}}\left(\Omega \right)=\frac{1}{\sqrt{2\pi }}e^{im\theta_{D-1}}\prod_{j=1}^{D-2}{\hat{C}}_{\mu_{j}-\mu_{j+1}}^{\left(\alpha_{j}+\mu_{j+1}\right)}\left(\cos\theta_{j}\right){\left(\sin\theta_{j}\right)}^{\mu_{j+1}},$$

with $2\alpha_{j}=D-j-1$ and ${\hat{C}}_{n}^{\left(\lambda \right)}\left(x\right)$, $\lambda >-\frac{1}{2}$, denotes the (orthonormal) Gegenbauer or ultraspherical polynomial [84] of degree n and parameter λ which satisfies the orthonormality

(8) $$\int_{-1}^{1}{\hat{C}}_{n}^{\left(\lambda \right)}\left(x\right)\;\;{\hat{C}}_{m}^{\left(\lambda \right)}\left(x\right){\left(1-x^{2}\right)}^{\lambda -\frac{1}{2}}dx=\delta_{mn},$$

These angular hyperfunctions form an orthonormal set which satisfies the orthonomalization condition

(9) $$\int_{S_{D-1}}Y_{l^{\prime },\left\{\mu^{\prime }\right\}}^{*}\left(\Omega_{D-1}\right)\;\;Y_{l,\left\{\mu \right\}}\left(\Omega_{D-1}\right)d\Omega_{D-1}=\delta_{l,l^{\prime }}\delta_{\left\{\mu \right\},\left\{\mu^{\prime }\right\}}$$

so that

(10) $$\int_{S_{D-1}}{\left|Y_{l,\{\mu \}}\left(\Omega_{D-1}\right)\right|}^{2}d\Omega_{D-1}=1.$$

Thus, they form a standard basis of the irreducible representations of the rotation group SO(D) in the space of the *D*-dimensional unit sphere with the invariant measure

$$d\Omega_{D-1}=\left(\prod_{j=1}^{D-2}{\left(\sin\theta_{j}\right)}^{2\alpha_{j}}d\theta_{j}\right)d\theta_{D-1}.$$

Then, by taking into account Equations (2) and (6), it turns out that the position eigenfunctions of the system are given by

(11) $$\begin{array}{ccc} \Psi_{n,l,\left\{\mu \right\}}\left(r\right) & = & R_{n,l}\left(r\right)\times Y_{l,\{\mu \}}\left(\Omega_{D-1}\right), \end{array}$$

where *n* denotes the radial hyperquantum number, and the angular part of the eigenfunctions are the hyperspherical harmonics $Y_{l,\{\mu \}}\left(\Omega_{D-1}\right)$. The radial position eigenfunction $R_{n,l}\left(r\right)$ is known to fulfill the radial equation [21,85]

(12) $$\left[-\frac{1}{2}\frac{d^{2}}{dr^{2}}-\frac{D-1}{2r}\frac{d}{dr}+\frac{l(l+D-2)}{2r^{2}}+V_{D}\left(r\right)\right]R_{nl}\left(r\right)=E_{nl}\;R_{nl}\left(r\right).$$

It is often convenient to make the change of variable $R_{n,l}\left(r\right)\;\to \;u_{n,l}\left(r\right):=r^{\frac{D-1}{2}}R_{n,l}\left(r\right)$, since then this equation transforms into the reduced radial Schrödinger equation

(13) $$\left[-\frac{1}{2}\frac{d^{2}}{dr^{2}}+V_{eff}\right]u_{n,l}\left(r\right)=E_{nl}\;u_{n,l}\left(r\right),$$

with the effective potential

(14) $$V_{eff}\left(r\right)=V_{D}\left(r\right)+\frac{L(L+1)}{2r^{2}}=V_{D}\left(r\right)+\frac{l(l+D-2)}{2r^{2}}+\frac{\left(D-1)(D-3\right)}{8r^{2}}$$

(with the grand quantum orbital quantum number $L=l+\frac{D-3}{2}$), which can be also expressed as

(15) $$V_{eff}\left(r\right)=V_{D}\left(r\right)+\frac{{\left(D+2l\right)}^{2}-4\left(D+2l\right)+3}{8r^{2}}$$

This Schrödinger equation describes the one-dimensional non-relativistic movement of the particle subject to the natural force coming from the external potential $V_{D}\left(r\right)$ and two additional forces with different physical origin: the centrifugal force associated with a non-vanishing hyperangular momentum, and a quantum fictitious force associated to the quantum-centrifugal potential $\frac{1}{4}\left(D-1\right)\left(D-3\right)$ of purely dimensional origin (since it emerges directly from acting with the D-dimensional Laplacian on the wavefunction (11)). The latter potential vanishes for D=1 and D=3, and it is negative for D=2 and positive for D≥4. Moreover, the quantum fictitious force exists irrespective of the hyperangular momentum and has a quadratic dependence on the dimensionality, being attractive (repulsive) for D=2 (D≥4); see [86,87,88,89,90] for its relevant physical effects. Besides, according to Equation (15), the effective potential $V_{eff}$ depends on *D* and *l* through a special (D+2l)-combination which explains the Van Vleck’s interdimensional-degeneracy phenomenon [91]; this implies e.g., that for an arbitrary potential the energies of the 7-dimensional *s* states are the same as those of the 5-dimensional *p* states or the 3-dimensional *d* states [6,92].

It is worth to highlight that the *D*-dimensional Schrödinger equation is formally the same as the three-dimensional one but with the grand orbital quantum number $L=l+\frac{D-3}{2}$. This shows the existence of an isomorphism [6,93] between the dimensionality and the hyperangular quantum number, so that D→D+2 is equivalent to l→l+1. Note, in addition, that the physical solutions of the Schrödinger Equation (9) requires that u(r) tends to zero when r goes to zero and to infinity, and it fulfills

(16) $$\int_{0}^{\infty }{\left|u_{n,l}\left(r\right)\right|}^{2}dr=1,$$

in order that the wavefunctions to be duly normalized: $\int {\left|\Psi_{n,l,\left\{\mu \right\}}\left(r\right)\right|}^{2}dr=1$, with the *D*-dimensional volume element $dr=r^{D-1}dr\;d\Omega_{D-1}$; keep in mind the normalization to unity of the hyperspherical harmonics given by Equation (10). Thus, the physical wavefunctions in the hyperspherical coordinate system $\left(r,\theta_{1},\theta_{2},\ldots ,\theta_{D-1}\right)$ are described by the *D* hyperquantum integer numbers $\left(n,l,\left\{\mu \right\}\right)\equiv \left(n,\mu_{1},\mu_{2},\ldots ,\mu_{D-1}\right)$.

In momentum space the wavefunctions for a generic stationary state (n,l,{μ}) of the *D*-dimensional system are given by $\tilde{\psi }\left(p,t\right)=\tilde{\Psi }\left(p\right)\;e^{-iEt}$, where the momentum eigenfunction ${\tilde{\Psi }}_{n,l,\{\mu \}}\left(p\right)$ can be obtained by means of the Fourier transform of the position eigenfunction (11),

(17) $${\tilde{\Psi }}_{n,l,\{\mu \}}\left(p\right)=\int_{R_{D}}e^{-ip\cdot r}\;\Psi_{n,l,\left\{\mu \right\}}\left(r\right)\;dr,$$

giving rise to

(18) $${\tilde{\Psi }}_{n,l,\{\mu \}}\left(p\right)=M_{n,l}\left(p\right)\times Y_{l,\{\mu \}}\left(\Omega_{D-1}\right),$$

where $p=(p,\theta_{1},\ldots ,\theta_{D-1})$, and the radial momentum eigenfunction $M_{n,l}\left(p\right)$ of the system is related to the radial position eigenfunction $R_{n,l}\left(r\right)$ through the Hankel transform

(19) $$M_{n,l}\left(p\right)=p^{1-\frac{D}{2}}\int_{0}^{+\infty }r^{\frac{D}{2}}R_{n,l}\left(r\right)\;J_{l+\frac{D}{2}-1}\left(pr\right)\;dr,$$

where $J_{\nu }\left(pr\right)$ is the Bessel function of the first kind with order ν [84]. Finally, note that $\int {\left|\Psi_{n,l,\left\{\mu \right\}}\left(p\right)\right|}^{2}dp=1$, with the generalized solid angle element $dp=p^{D-1}dp\;d\Omega_{D-1}$.

### 2.2. The Probability Densities

The position and momentum probability densities of the *D*-dimensional stationary state (n,l,{μ}) are given by the modulus squared of the two corresponding eigenfunctions as

(20) $$\begin{array}{ccc} \rho_{n,l,\{\mu \}}\left(r\right) & = & {\left|\Psi_{n,l,\{\mu \}}\left(r\right)\right|}^{2}=|R_{nl}{\left(r\right)|}^{2}\times {\left|Y_{l,\{\mu \}}\left(\Omega_{D-1}\right)\right|}^{2} \end{array}$$

(21) $$\begin{array}{ccc} & = & r^{1-D}\;|u_{nl}{\left(r\right)|}^{2}\times {\left|Y_{l,\{\mu \}}\left(\Omega_{D-1}\right)\right|}^{2} \end{array}$$

in position space, and

(22) $$\begin{array}{ccc} \gamma_{n,l,\{\mu \}}\left(p\right) & = & {\left|{\tilde{\Psi }}_{n,l,\{\mu \}}\left(p\right)\right|}^{2}={\left|M_{n,l}\left(p\right)\right|}^{2}\times {\left|Y_{l,\{\mu \}}\left({\hat{\Omega }}_{D-1}\right)\right|}^{2} \end{array}$$

(23) $$\begin{array}{ccc} & = & p^{1-D}\;|{\tilde{u}}_{nl}{\left(p\right)|}^{2}\times {\left|Y_{l,\{\mu \}}\left(\Omega_{D-1}\right)\right|}^{2} \end{array}$$

in momentum space, where the reduced radial momentum eigenfunction.

(24) $${\tilde{u}}_{nl}\left(p\right)={\left(-i\right)}^{l}\int_{0}^{\infty }\sqrt{r\;p}\;\;J_{l+D/2-1}\left(r\;p\right)\;u\left(r\right)dr,$$

is the Hankel transform of the reduced radial position eigenfunction $u_{nl}\left(r\right)$.

Here, we are interested in the spatial extension of the electronic position and momentum densities of a *D*-dimensional single-particle system far beyond the variance. This will be done in the following chapters by means of the dispersion measures (the radial and logarithmic expectation values), the entropy-like measures of local and global character (e.g., Fisher’s information, Shannon’s and Rényi’s entropies).

## 3. Dispersion Measures and Heisenberg-Like Uncertainty for Central Potentials

In this section we first give a number of universally-valid inequality-based properties fulfilled by the dispersion measures (i.e., the radial and logarithmic expectation values) of general *D*-dimensional single-particle systems in position and momentum spaces, and then we study how they get modified for the systems with an arbitrary central potential $V_{D}\left(r\right)$. The effects of the spherical symmetry of the potential on these quantities and their properties are explicitly shown by their dependence on the angular hyperquantum numbers (l,{μ}) of the system’s states.

The radial and logarithmic expectation values of the *D*-dimensional probability densities of the stationary state (n,l,{μ}) can be expressed, keeping in mind Equations (20) and (22), as

(25) $$\begin{array}{ccc} \langle r^{\alpha }\rangle & := & \int_{R_{D}}r^{\alpha }\rho_{n,l,\{\mu \}}\left(\vec{r}\right)d\vec{r}=\int_{0}^{\infty }r^{\alpha +D-1}{\left|R_{n,l}\left(r\right)\right|}^{2}dr \end{array}$$

(26) $$\langle \ln r\rangle =\int \left(\ln r\right)\rho_{n,l,\{\mu \}}\left(\vec{r}\right)\;d\vec{r}=\int_{0}^{\infty }\left(\ln r\right)r^{D-1}{\left|R_{n,l}\left(r\right)\right|}^{2}dr$$

in position space, and

(27) $$\begin{array}{ccc} \langle p^{\alpha }\rangle & := & \int p^{\alpha }\gamma_{n,l,\{\mu \}}\left(\vec{p}\right)d\vec{p}=\int_{0}^{\infty }p^{\alpha +D-1}{\left|M_{n,l}\left(p\right)\right|}^{2}dp \end{array}$$

(28) $$\langle \ln p\rangle =\int \left(\ln p\right)\gamma_{n,l,\{\mu \}}\left(\vec{p}\right)\;d\vec{p}=\int_{0}^{\infty }\left(\ln p\right)p^{D-1}{\left|M_{n,l}\left(p\right)\right|}^{2}dp$$

in momentum space. For the second equality we have used the normalization condition (10) of the hyperspherical harmonics. These radial and logarithmic quantities are often accessible, at least for D=3, e.g., by electron scattering and from the experimental Compton profile in position and momentum spaces [94,95] (see also [96] for further references), respectively. We cannot explicitly calculate these quantities because we do not know the analytical expression of the corresponding radial eigenfunctions $R_{n,l}\left(r\right)$ and $M_{n,l}\left(p\right)$. However, these spreading measures have a number of inequality-based properties. For example, Ray et al. [85] found the following three-term recurrence relation

(29) $$\begin{array}{c} {\left(2L+1\right)}^{2}C_{n,l}^{2}\;\delta_{\alpha ,-2L}=\frac{\alpha -1}{2}\left[{\left(2L+1\right)}^{2}-{\left(\alpha -1\right)}^{2}\right]\langle r^{\alpha -3}\rangle \\ \;+2\;[\langle r^{\alpha }V^{\prime }\rangle +2\alpha \langle r^{\alpha -1}V\rangle -2\alpha \;E_{n,l}\;\langle r^{\alpha -1}\rangle ], \end{array}$$

(which holds for α≥2L), where $V^{\prime }=\frac{dV}{dr}$, $r^{2}V\left(r\right)\to 0$ when r→0, and $C_{n,l}$ denotes the normalization constant of the radial eigenfunction, which fulfills that $C_{n,l}=\lim_{r\to 0}r^{-l}R_{n,l}=\frac{1}{l!}\frac{d^{l}R_{n,l}}{d\;r^{l}}$. In particular, $C_{0}=R_{n,0}\left(0\right)$ is the value of the radial eigenfunction at the origin. Similar two- and three-term recursion results (and some extensions to off-diagonal radial matrix elements) have been obtained by Dong et al. [13,97]; in particular they have found that

(30) $$\langle V^{\prime }\rangle =L\left(L+1\right)\;\langle r^{-3}\rangle$$

Explicit expressions cannot be obtained unless the analytical form of the potential V(r) is given, such as e.g., the oscillator-like quadratic and Coulomb-like hydrogenic potentials [96,98].

However, a number of uncertainty-like inequalities fulfilled by the dispersion quantities (25)–(28) have been derived by a variety of methods. Angulo [99], by means of the maximization method [100] for the position and momentum Shannon entropies of the system in terms of the radial expectation values, found the inequalities

(31) $${\langle r^{a}\rangle }^{\frac{2}{a}}{\langle p^{b}\rangle }^{\frac{2}{b}}\geq \;D\left(a,b\right)\;=\;\left(\frac{e\;D^{\frac{2}{a}}\;\Gamma^{\frac{2}{D}}\left(1+\frac{D}{2}\right)}{{\left(ae\right)}^{\frac{2}{a}}\;\Gamma^{\frac{2}{D}}\left(1+\frac{D}{a}\right)}\right)\;\left(\frac{e\;D^{\frac{2}{b}}\;\Gamma^{\frac{2}{D}}\left(1+\frac{D}{2}\right)}{{\left(be\right)}^{\frac{2}{b}}\;\Gamma^{\frac{2}{D}}\left(1+\frac{D}{b}\right)}\right)$$

(for a,b≥0) which are universally valid (i.e., they hold for all *D*-dimenional quantum systems); they simplify for a=b=2 to the familiar *D*-dimensional form of Heisenberg inequality [101,102]

(32) $$\langle r^{2}\rangle \langle p^{2}\rangle \geq \frac{D^{2}}{4}$$

Later, Zozor et al. [50] has improved the uncertainty inequality (31) by using a similar approach based on the Rényi-entropies, obtaining that

(33) $${\langle r^{a}\rangle }^{\frac{2}{a}}\;{\langle p^{b}\rangle }^{\frac{2}{b}}\;\geq \;C\left(a,b\right)\;=\;\max_{\alpha \in D}\;B\left(\alpha \right)\;M\left(a,\alpha \right)\;M\left(b,\alpha^{*}\right)$$

where $\alpha^{*}=\alpha /\left(2\alpha -1\right)$, $D=\left(\max\left(\frac{1}{2}\;,\;\frac{D}{D+a}\right)\;;\;1\right],$ and the functions B(α) and M are given by

(34) $$B\left(\alpha \right)=\frac{\alpha^{\frac{1}{\alpha -1}}\alpha^{*\;\frac{1}{\alpha^{*}-1}}}{4e^{2}}\;\mathrm{for}\;\alpha \neq 1\;\mathrm{and}\;B\left(1\right)=\frac{1}{4}\;,$$

and

(35) $$M\left(l,\lambda \right)=\left\{\begin{array}{cc} \;\;2\pi e\;{\left(\;\frac{l}{\Omega B\left(\frac{D}{l},1-\frac{\lambda }{\lambda -1}-\frac{D}{l}\;\right)}\right)}^{\;\;\frac{2}{D}}\;{\left(\frac{D\;(1-\lambda )}{D(\lambda -1)+l\lambda }\right)}^{\;\;\frac{2}{l}}{\left(\frac{l\lambda }{D(\lambda -1)+l\lambda }\right)}^{\;\;\frac{2}{D(\lambda -1)}}, & \;\\ \; & \;1-\frac{l}{l+D}<\lambda <1\;\\ \;\;2\pi e\;{\left(\frac{l}{\Omega \;\Gamma \left(\frac{D}{l}\right)}\right)}^{\;\;\frac{2}{D}}{\left(\frac{D}{le}\right)}^{\;\;\frac{2}{l}}, & \;\lambda =1\;\\ \;\;2\pi e\;{\left(\frac{l}{\Omega \;B\left(\;\frac{D}{l},\frac{\lambda }{\lambda -1}\;\right)}\right)}^{\;\;\frac{2}{D}}\;{\left(\frac{D(\lambda -1)}{D(\lambda -1)+l\lambda }\right)}^{\;\;\frac{2}{\lambda }}{\left(\frac{l\lambda }{D(\lambda -1)+l\lambda }\right)}^{\;\;\frac{2}{D(\lambda -1)}}, & \;\lambda >1 \end{array}\right.$$

respectively, where $\Omega =\frac{2\pi^{d/2}}{\Gamma (d/2)}$ and B(x,y) denotes the beta function [84]. The case b≥a>0 can be treated by means of the symmetry,

(36) $$\arg\max_{\alpha }B\left(\alpha \right)\;M\left(a,\alpha \right)\;M\left(b,\alpha^{*}\right)=\alpha_{\mathrm{opt}}\left(a,b\right)={\left(\alpha_{\mathrm{opt}}\left(b,a\right)\right)}^{*}$$

and C(b,a)=C(a,b). The $\alpha_{\mathrm{opt}}$-symmetry provokes that $\alpha_{\mathrm{opt}}\left(a,a\right)=1$ and thus the optimal bound from this Rényi-entropy-based approach coincides with the Shannon-entropy-based bound D(a,b) given by (31) in this case. Unfortunately, except for the case a=b, it does not seem possible to give an analytical expression for C(a,b). Let us also point out that this information-theoretical formulation suffers from the fact that the bound is probably not sharp, and the analytical determination of the minimizers requires a variational approach, not yet done.

In addition, from the Pitt-Beckner inequality [103,104] we can also show that the expectation values $\left(\langle p^{\alpha }\rangle ,\langle r^{-\alpha }\rangle \right)$ satisfy the uncertainty relations [11]

(37) $$\langle p^{\alpha }\rangle \geq 2^{\alpha }{\left[\frac{\Gamma \left(D+\frac{\alpha }{4}\right)}{\Gamma \left(D-\frac{\alpha }{4}\right)}\right]}^{2}\langle r^{-\alpha }\rangle ;\;0\leq \alpha <D$$

which for α=2 boils down to

(38) $$\langle p^{2}\rangle \geq {\left(\frac{D-2}{2}\right)}^{2}\langle r^{-2}\rangle ;\;D>2.$$

Moreover, we also have the reciprocal uncertainty-like inequality

(39) $$\langle r^{\alpha }\rangle \geq 2^{\alpha }{\left[\frac{\Gamma \left(D+\frac{\alpha }{4}\right)}{\Gamma \left(D-\frac{\alpha }{4}\right)}\right]}^{2}\langle p^{-\alpha }\rangle ;\;0\leq \alpha <D$$

which extends a number of relations previously found [105,106]; and in the limit α→0 it supplies the Beckner’s logarithmic uncertainty sum [104]

(40) $$\langle \ln r\rangle +\langle \ln p\rangle \geq \psi \left(\frac{D}{4}\right)+\ln2$$

where $\psi \left(x\right)=\Gamma^{\prime }\left(x\right)/\Gamma \left(x\right)$ is the psi or digamma function. This uncertainty sum is related to the logarithmic uncertainty product by means of the logarithmic uncertainty relation [101]

(41) $$\Delta \left(\ln r\right)\Delta \left(\ln p\right)\geq \frac{\Gamma^{2}\left(\frac{D}{2}\right)}{8\pi }\exp\left[D-1-D\left(\langle \ln r\rangle +\langle \ln p\rangle \right)\right],$$

(proved in Section 4) where the logarithmic standard deviation is given as

(42) $$\Delta \left(\ln r\right)\equiv {\left(\langle {\left(\ln r\right)}^{2}\rangle -{\langle \ln r\rangle }^{2}\right)}^{1/2},$$

which illustrates the uncertainty character of the logarithmic sum. In addition, note that the product of (37) and (39) gives rise to the inequality relation

(43) $$\langle r^{\alpha }\rangle \langle p^{\alpha }\rangle \geq 2^{2\alpha }{\left[\frac{\Gamma \left(D+\frac{\alpha }{4}\right)}{\Gamma \left(D-\frac{\alpha }{4}\right)}\right]}^{4}\langle r^{-\alpha }\rangle \langle p^{-\alpha }\rangle$$

The universally valid uncertainty inequalities (31)–(43) can be improved for central potentials as follows. A first way of improvement comes from the observation that for these potentials we have [107,108] that

(44) $$\begin{array}{ccc} \langle p^{2}\rangle & = & J_{R}\left(D\right)+l\left(l+D-2\right)\langle r^{-2}\rangle \end{array}$$

The symbol $J_{R}\left(D\right)$ denotes the radial integral

(45) $$J_{R}\left(D\right):=\int_{0}^{\infty }{\left[\frac{dR_{nl}\left(r\right)}{dr}\right]}^{2}r^{D-1}dr=\int_{0}^{\infty }{\left[\frac{du_{nl}\left(r\right)}{dr}\right]}^{2}dr+\frac{1}{4}\left(D-1\right)\left(D-3\right)\langle r^{-2}\rangle ,$$

where we have used the expression

(46) $$\int_{0}^{\infty }r^{-1}u_{nl}\left(r\right)\frac{du_{nl}\left(r\right)}{dr}dr=\frac{1}{2}\int_{0}^{\infty }r^{-2}{\left[u_{nl}\left(r\right)\right]}^{2}dr$$

and we have assumed that $u_{nl}\left(r\right)∼r^{L+1}$ for r→0; the latter occurs for $r^{2}V\left(r\right)\to 0$ when r→0, and implies that D≥2. Note that Equation (44) and the non-negativity of $J_{R}\left(D\right)$ allows one to obtain the inequality

(47) $$\langle p^{2}\rangle \geq l\left(l+D-2\right)\;\langle r^{-2}\rangle =\left[L\left(L+1\right)-\frac{1}{4}\left(D-1\right)\left(D-3\right)\right]\langle r^{-2}\rangle$$

valid for D≥2; and $\langle p^{2}\rangle \geq m^{4}\;\langle r^{-2}\rangle$ for D=2. Then, the reciprocity of the position and momentum spaces allows us to obtain the conjugate inequality

(48) $$\langle r^{2}\rangle \geq \left[L\left(L+1\right)-\frac{1}{4}\left(D-1\right)\left(D-3\right)\right]\;\langle p^{-2}\rangle$$

and $\langle r^{2}\rangle \geq m^{4}\;\langle p^{-2}\rangle$ for D=2. Multiplying (47) and (48) one has the following relation between the Heisenberg-like uncertainty products $\langle r^{2}\rangle \langle p^{2}\rangle$ and $\langle r^{-2}\rangle \langle p^{-2}\rangle$:

(49) $$\langle r^{2}\rangle \langle p^{2}\rangle \geq {\left[L\left(L+1\right)-\frac{1}{4}\left(D-1\right)\left(D-3\right)\right]}^{2}\;\langle r^{-2}\rangle \langle p^{-2}\rangle ,\;\mathrm{for}\;D\geq 2$$

Similarly, from Equations (44) and (45), one has that

(50) $$\begin{array}{ccc} \langle p^{2}\rangle & = & \int_{0}^{\infty }{\left[\frac{du_{nl}\left(r\right)}{dr}\right]}^{2}dr\;+\;L\left(L+1\right)\langle r^{-2}\rangle , \end{array}$$

which, together with the nonnegativity of this integral functional, gives rise to the inequality

(51) $$\langle p^{2}\rangle \geq L\left(L+1\right)\langle r^{-2}\rangle ,\;\mathrm{for}\;D\geq 3$$

and reciprocally,

(52) $$\langle r^{2}\rangle \geq L\left(L+1\right)\langle p^{-2}\rangle ,\;\mathrm{for}\;D\geq 3,$$

so that it is fulfilled that

(53) $$\langle r^{2}\rangle \langle p^{2}\rangle \geq {\left[L(L+1)\right]}^{2}\;\langle r^{-2}\rangle \langle p^{-2}\rangle ,\;\mathrm{for}\;D\geq 3$$

which improves the uncertainty relations (47)–(49), respectively.

A further improvement for central potentials is obtained [11] by taking into account (50) and optimizing the inequality

(54) $$\int_{0}^{\infty }{\left(\frac{du_{nl}\left(r\right)}{dr}-\lambda \;r^{\beta }u_{nl}\left(r\right)\right)}^{2}dr\geq 0$$

with respect to λ. Then, one obtains [11] that

(55) $$\langle p^{2}\rangle \geq L\left(L+1\right)\langle r^{-2}\rangle +\frac{\beta^{2}}{4}\frac{{\langle r^{\beta -1}\rangle }^{2}}{\langle r^{2\beta }\rangle }$$

with $\beta >-L-\frac{3}{2}$. Then, for β=−1 one obtains that

(56) $$\langle p^{2}\rangle \geq {\left(L+\frac{1}{2}\right)}^{2}\;\langle r^{-2}\rangle ={\left(l+\frac{D-2}{2}\right)}^{2}\;\langle r^{-2}\rangle$$

which improves the universally-valid inequality (38) for any specific value of the orbital hypyerquamtum number *l*, and improves the central-potential inequality (47) (and so, (51) once again). The reciprocal expression

(57) $$\langle r^{2}\rangle \geq {\left(L+\frac{1}{2}\right)}^{2}\;\langle p^{-2}\rangle ={\left(l+\frac{D-2}{2}\right)}^{2}\;\langle p^{-2}\rangle$$

is also fulfilled, which generalizes the inequalities (48) and (52) in a similar manner. Moreover, by working out similarly, it also happens for central potentials [11] that

(58) $$\langle p^{2}\rangle \geq \frac{1}{4}{\left(2L+\beta +2\right)}^{2}\;\frac{{\langle r^{\beta -1}\rangle }^{2}}{\langle r^{2\beta }\rangle }$$

with $\beta >-L-\frac{3}{2}$ and D>1−β. Then, for β=0 and 1 one obtains that

(59) $$\langle p^{2}\rangle \geq {\left(L+1\right)}^{2}{\langle r^{-1}\rangle }^{2}={\left(l+\frac{D-1}{2}\right)}^{2}{\langle r^{-1}\rangle }^{2}$$

and [109]

(60) $$\langle r^{2}\rangle \langle p^{2}\rangle \geq {\left(L+\frac{3}{2}\right)}^{2}={\left(l+\frac{D}{2}\right)}^{2},$$

respectively. The last two inequalities extend and improve the universally-valid relation $\langle p^{2}\rangle \geq {\left(\frac{D-1}{2}\right)}^{2}{\langle r^{-1}\rangle }^{2}$ of Bialynicki-Birula et al. [89], and the Heisenberg uncertainty relation (49) to all excited stationary states of *D*-dimensional central potentials, respectively. The expression (60) is the best Heisenberg position-momentum uncertainty relation for central potentials known up until now. See also [110,111] for three-dimensional systems. Moreover, many other uncertainty-like relations encountered for three-dimensional systems are particular instances of the previous general inequalities (55) and (58) for *D*-dimensional systems (see e.g., [104,112,113,114,115,116].

In addition, again with the reciprocity property of the position and momentum spaces, the conjugate expressions of the inequalities (55) and (58) straightforwardly follow; for example we have that

(61) $$\langle r^{2}\rangle \geq \frac{1}{4}{\left(2L+\beta +2\right)}^{2}\;\frac{{\langle p^{\beta -1}\rangle }^{2}}{\langle p^{2\beta }\rangle }$$

for D>1−β and $\beta >-L-\frac{3}{2}$; for β=0 and 1 and D>2 we have the expression (60) and the inequality

(62) $$\langle r^{2}\rangle \geq {\left(L+1\right)}^{2}\;{\langle p^{-1}\rangle }^{2},$$

respectively, which generalize to *D* dimensions various similar three-dimensional inequalities (see e.g., [112,113,116,117]).

Let us now examine the spherical-symmetry effects on the Beckner’s logarithmic-sum uncertainty (40). Rudnicki et al. [118] have rigorously shown by means of the Omri’s integral inequality [119] that the Beckner’s relation (40) gets improved as

(63) $$\langle \ln r\rangle +\langle \ln p\rangle \geq \psi \left(\frac{D+2l}{4}\right)+\ln2,\;l=0,1,2,\ldots$$

for central potentials. This inequality allows us not only to improve the logarithmic uncertainty relation (41) based on the logarithmic standard deviation as

(64) $$\Delta \left(\ln r\right)\;\;\Delta \left(\ln p\right)\geq \frac{\Gamma^{2}\left(\frac{D}{2}\right)}{8\pi }\exp\left[D\left(1-\ln2\right)-1-D\;\psi \left(\frac{D+2l}{4}\right)\right]$$

but also to obtain the Shannon-entropy-based uncertainty relation for central potentials in the next section.

Finally, the previous uncertainty inequalities can be still improved for various broad relevant classes of central potentials such as e.g., the convex potentials [120,121,122,123] and the power-law or anharmonic potentials [124,125,126,127,128,129]. This improvement, however, has not yet been done up until now. Explicit values for the radial and logarithmic expectation values have been recently obtained for a few central potentials, such as the hydrogenic-like and the oscillator-like potentials (see e.g., [98,129]).

## 4. Information–Theoretical Measures and Entropic Uncertainty for Central Potentials

In this section we discuss the position and momentum information-theoretical entropies of Fisher, Shannon and Rényi types (and their associated uncertainty relations) for ground and excited states (n,l,{μ}) of arbitrary *D*-dimensional central potentials. They are denoted by $\left(F\left[\rho_{n,l,\{\mu \}}\right],S\left[\rho_{n,l,\{\mu \}}\right],R_{q}\left[\rho_{n,l,\{\mu \}}\right]\right)$ and $\left(F\left[\gamma_{n,l,\{\mu \}}\right],S\left[\gamma_{n,l,\{\mu \}}\right],R_{q}\left[\gamma_{n,l,\{\mu \}}\right]\right)$, respectively, where we should keep in mind the expressions (20) and (22) of the quantum densities of the state in the two conjugated spaces. For each information-theoretical measure of the multidimensional quantum states we start giving the universally-valid upper and lower bounds in terms of the radial expectation values and the associated uncertainty relation. Then we show and discuss the improvement of these inequality-based properties for the systems with a central potential by giving their explicit dependence on the state’s angular hyperquantum numbers. The dependence on the radial hyperquantum number cannot be given, because the radial eigenfunction is not known since the analytical form of the central potential is assumed to be unknown.

These uncertainty measures are the basic variables of the information theory of quantum systems which constitutes the fundamental pillar of the classical and quantum information and computation [24,26,37,38]. They quantify the global and local spreading of the charge and momentum of the system along the domain of definition of the spherically-symmetric potential in a much better way that the dispersion measures considered in the previous section. This is partially because the dispersion measures are measures of separation of the region(s) of concentration of the quantum probability cloud from a specific point of the distribution, rather than measures of the extent to which the distribution is in fact concentrated [130]. Information theory [24,26] provides more appropriate local (Fisher information) and global (e.g., Shannon, Rényi and Tsallis entropies) uncertainty measures which do not depend on any particular point of the multidimensional domain of the density.

The Fisher information [22,24] is a local uncertainty measure which quantifies the gradient content of the density, so that it is very sensitive to the fluctuations of the density. The Shannon and Rényi entropies [25,27,28] are global uncertainty measures which quantify the various macroscopic, observable aspects of the spatial extension of the density [39,74].

### 4.1. The Fisher Informations

Here, we begin with the definition of the Fisher information of a multidimensional quantum state in both position and momentum spaces. Then, we give and discuss their upper and lower bounds of universal validity in terms of the position and momentum Heisenberg-like measures. Later the Fisher informations for the quantum states of arbitrary central potentials are considered, and their explicit expressions in position and momentum spaces are given in terms of pairs of radial expectation values $\left(\langle p^{2}\rangle ,\langle r^{-2}\rangle \right)$ and $\left(\langle r^{2}\rangle ,\langle p^{-2}\rangle \right)$, respectively. Finally, the uncertainty character for the product of the position and momentum Fisher informations is shown for central potentials. Moreover, the associated uncertainty relation is rigorously proved for central potentials and for general *D*-dimensional quantum states with real-valued position (or momentum) wavefunctions.

The Fisher information for a *D*-dimensional quantum state with the position probability density ρ(r) is defined [24] by

(65) $$\begin{array}{ccc} F[\rho ] & := & \int_{R_{D}}\frac{|{\vec{\nabla }}_{D}{\;\rho \left(r\right)|}^{2}}{\rho (r)}\;dr=4\int_{R_{D}}{\left[{\vec{\nabla }}_{D}\sqrt{\rho (r)}\right]}^{2}dr. \end{array}$$

This quantity, which is closely related [64,65] to the Weizsäcker energy $T_{W}\left[\rho \right]=\frac{1}{8}F\left[\rho \right],$ is a local spreading measure of the state’s density so that it quantifies the pointwise concentration of ρ. Then, the Fisher information controls the localization of the density around its nodes, appropriately grasping the oscillatory nature of the wavefunctions of the quantum-mechanical states. This confers it a relevant role in the characterization of numerous scientific phenomena of standard and non-standard *D*-dimensional systems. Moreover, it describes a local uncertainty measure so that the higher this quantity is, the more localized is the density, the smaller is the uncertainty and the higher is the accuracy in predicting the localization of the particle. The corresponding quantity for the momentum density γ(p) is the momentum Fisher information defined as

(66) $$\begin{array}{ccc} F[\gamma ] & := & \int_{R_{D}}\frac{|{\vec{\nabla }}_{D}{\gamma \left(p\right)|}^{2}}{\gamma (p)}\;dp=4\int_{R_{D}}{\left[{\vec{\nabla }}_{D}\sqrt{\rho (p)}\right]}^{2}dp. \end{array}$$

First, these quantities have been found to be bounded from above [131,132] by the expectation values $\left(\langle r^{2}\rangle ,\langle p^{-2}\rangle \right)$ by the Stam uncertainty inequalities

(67) $$F\left[\rho \right]\leq 4\langle p^{2}\rangle ,\;F\left[\gamma \right]\leq 4\langle r^{2}\rangle$$

for general quantum systems. Second, the position Fisher information of general *D*-dimensional quantum systems has been variationally shown [65] to be bounded from below as

(68) $$F\left[\rho \right]\geq \frac{D^{2}}{\langle r^{2}\rangle }$$

and also as

(69) $$F\left[\rho \right]\geq {\left(D-1\right)}^{2}{\langle r^{-1}\rangle }^{2};\;F\left[\rho \right]\geq {\left(D-2\right)}^{2}\langle r^{-2}\rangle$$

Note that expression (68) is the celebrated Crámer-Rao inequality [26,133], valid for all the stationary states of arbitrary *D*-dimensional quantum systems, which saturates (i.e., equality is reached) [134] for the density associated with the ground state of the harmonic oscillator in $R_{D}$. When the domain of the density ρ(r) is bounded, the minimum value of the Fisher information is achieved by the ground state of the quantum box described itself by this domain [134]. The last three inequalities are instances of the general bounds [105,106]

(70) $$F\left[\rho \right]\geq {\left(\beta +D-1\right)}^{2}\frac{{\langle r^{\beta -1}\rangle }^{2}}{\langle r^{2\beta }\rangle },\;\mathrm{for}\;\beta \geq \max\left\{-D+1,-1\right\}$$

and

(71) $$F\left[\rho \right]\geq \langle r^{-2}\rangle \left[{\left(D-2\right)}^{2}+\frac{{\left(\beta +1\right)}^{2}{\langle r^{\beta -1}\rangle }^{2}}{\langle r^{2\beta }\rangle \langle r^{-2}\rangle -{\langle r^{\beta -1}\rangle }^{2}}\right],\;\beta \geq -1$$

in terms of the two and three radial expectation values $\langle r^{\alpha }\rangle$, respectively. These general expressions, derived by means of Redheffer’s integral inequalities of Weyl type [135], extend to *D* dimensions numerous inequalities obtained in the literature (see e.g., [113]).

Third, for central potentials the position and momentum densities of the *D*-dimensional state (n,l,{μ}) are given by $\rho \left(r\right)=\rho_{n,l,\{\mu \}}\left(r\right)$ and $\gamma \left(p\right)=\gamma_{n_{r},l,\left\{\mu \right\}}\left(p\right)$, according to the expressions (20) and (22), respectively. They can be decomposed into two radial and angular parts according to Equations (20) and (22), so that we can show that the corresponding Fisher informations factorize [107] as

(72) $$\begin{array}{ccc} F\left[\rho_{n,l,\{\mu \}}\right] & = & 4\int_{0}^{\infty }{\left[R_{nl}^{{}^{\prime }}\left(r\right)\right]}^{2}r^{D-1}dr+\langle r^{-2}\;\rangle F\left[Y_{l,\{\mu \}}\right]\\ & = & 4\langle p^{2}\rangle +\langle r^{-2}\;\rangle F\left[Y_{l,\{\mu \}}\right] \end{array}$$

and

(73) $$\begin{array}{ccc} F\left[\gamma_{n,l,\{\mu \}}\right] & = & 4\int_{0}^{\infty }{\left[M_{nl}^{{}^{\prime }}\left(p\right)\right]}^{2}p^{D-1}dp+\langle p^{-2}\rangle F\left[Y_{l,\{\mu \}}\right]\\ & = & 4\langle r^{2}\rangle +\langle p^{-2}\;\rangle F\left[Y_{l,\{\mu \}}\right], \end{array}$$

respectively, where the common symbol $F\left[Y;D\right]$ denotes the angular part given by

(74) $$\begin{array}{ccc} F\left[Y_{l,\{\mu \}}\right] & = & 4\sum_{i=1}^{D-2}\int_{S_{D-1}}{\left[\frac{\partial }{\partial \theta_{i}}Y_{l,\left\{\mu \right\}}\left(\theta_{1},\theta_{2},...,\theta_{D-2},0\right)\right]}^{2}d\Omega_{D-1}=-2\left|m\right|\left(2l+D-2\right) \end{array}$$

in both spaces. Then, we have finally the elegant expressions

(75) $$F\left[\rho_{n,l,\{\mu \}}\right]=4\langle p^{2}\rangle -2\left|m\right|\left(2l+D-2\right)\langle r^{-2}\rangle$$

(76) $$F\left[\gamma_{n,l,\{\mu \}}\right]=4\langle r^{2}\rangle -2\left|m\right|\left(2l+D-2\right)\langle p^{-2}\rangle$$

for the position and momentum Fisher informations of *D*-dimensional central potentials in terms of the pairs of radial expectation values $\left(\langle p^{2}\rangle ,\langle r^{-2}\rangle \right)$ and $\left(\langle r^{2}\rangle ,\langle p^{-2}\rangle \right)$, respectively. See also [108] for the three-dimensional case and some hydrogenic and harmonic applications.

Now, from the last two expressions and the previous inequalities (56)–(57), it is straightforward to obtain the following uncertainty-like relations between the position (momentum) Fisher information and the momentum (position) second-order radial expectation value:

(77) $$F\left[\rho_{n,l,\{\mu \}}\right]\geq 4\left(1-\frac{2\left|m\right|}{2L+1}\right)\langle p^{2}\rangle$$

and

(78) $$F\left[\gamma_{n,l,\{\mu \}}\right]\geq 4\left(1-\frac{2\left|m\right|}{2L+1}\right)\langle r^{2}\rangle$$

Then, from the last two inequalities and the Stam ones given by (67), one can readily show that the lower and upper bounds for the position and momentum Fisher informations of any *D*-dimensional central potential are

(79) $$4\left(1-\frac{2\left|m\right|}{2L+1}\right)\langle p^{2}\rangle \leq F\left[\rho_{n,l,\{\mu \}}\right]\leq 4\langle p^{2}\rangle ,$$

and

(80) $$4\left(1-\frac{2\left|m\right|}{2L+1}\right)\langle r^{2}\rangle \leq F\left[\gamma_{n,l,\{\mu \}}\right]\leq 4\langle r^{2}\rangle ,$$

respectively.

Moreover, from relations (77)–(78) one has that the position and momentum Fisher informations of central potentials satisfy [107] the relation

(81) $$F\left[\rho_{n,l,\{\mu \}}\right]\times F\left[\gamma_{n,l,\{\mu \}}\right]\geq 16{\left[1-\frac{2\left|m\right|}{2L+1}\right]}^{2}\langle r^{2}\rangle \langle p^{2}\rangle ,$$

which is a clear manifestation of the uncertainty character of the Fisher-information product F[ρ]×F[γ]. Then, from Equations (60) and (81) we finally have the Fisher-information-based uncertainty relation [109]

(82) $$F\left[\rho_{n,l,\{\mu \}}\right]\times F\left[\gamma_{n,l,\{\mu \}}\right]\geq 16{\left[1-\frac{2\left|m\right|}{2L+1}\right]}^{2}\;{\left(L+\frac{3}{2}\right)}^{2}=16{\left[1-\frac{2\left|m\right|}{2l+D-2}\right]}^{2}\;{\left(l+\frac{D}{2}\right)}^{2},$$

which is valid for all wavefunctions of arbitrary central potentials. This relation extends and improves similar expressions previously obtained in three [108] and *D* [107] dimensions. Note that for *S* states (i.e., when l=0), this central-potential inequality boils down as $F\left[\rho_{n,0,\{0\}}\right]\times F\left[\gamma_{n,0,\{0\}}\right]\geq 4D^{2}$. Moreover, let us finally point out that the Fisher-information-based uncertainty relation (83)

(83) $$F\left[\rho \right]\times F\left[\gamma \right]\geq 4D^{2}$$

has been rigorously proved for general one-dimensional states with even real-valued wavefunctions [136] and for general *D*-dimensional quantum states with real-valued position (or momentum) wavefunctions [62]. However, this is not the most universal uncertainty relation expressible as a lower bound to the product of the Fisher measures F[ρ] and F[γ] because the Fisher product F[ρ]×F[γ] can be made arbitrarily small [137]. See also [138], where this problem has been discussed for pure and mixed states.

### 4.2. The Shannon Entropies

Here, we begin with the definition of the position and momentum Shannon entropies of a multidimensional quantum state; then, we show their universally-valid lower and upper bounds by means of the position and momentum Heisenberg-like and logarithmic measures, and the uncertainty character for the sum of the position and momentum Shannon entropies is shown. Later, the Shannon entropy for the quantum states of arbitrary central potentials are considered; it is explicitly shown how the spherical-symmetry effects of the quantum potential improve the associated position-momentum uncertainty relation and the upper bounds of the Shannon entropies in both conjugated spaces. Finally, some open problems are briefly discussed.

The Shannon entropy for a *D*-dimensional quantum state with the density ρ(r) is defined [25] by

(84) $$\begin{array}{ccc} S[\rho ] & := & -\int_{R_{D}}\rho \left(r\right)\ln\rho \left(r\right)\;dr, \end{array}$$

This quantity is a global spreading measure of the state’s density which does not depend on any particular point of its multidimensional domain. The Shannon entropy, closely connected to the thermodynamical entropy in the case of a thermal ensemble [139], fulfills all the hypotheses of Shannon theorem [25,26] and other important criteria [139,140]. It is worth noting that $S\left[\rho \right]$ can have any values in $\left[-\infty ,\infty \right]$, contrary to the differential Shannon entropy, $-\sum_{i}p_{i}\ln p_{i}$, of a probability on a discrete sample space which is always positive; moreover, $S\left[\rho \right]$ can also be undefined. Any sharp peaks in ρ(r) will tend to make S[ρ] negative, whereas positive values fo S[ρ] are provoked by a slowly decaying tail; hence, the Shannon entropy S[ρ] estimates the total multidimensional extent of the density ρ.

Similarly, the momentum entropy is given as

(85) $$\begin{array}{ccc} S[\gamma ] & := & -\int_{R_{D}}\gamma \left(r\right)\ln\gamma \left(r\right)\;dr, \end{array}$$

where γ(p) denotes the momentum density. These two position and momentum information entropies define uncertainty measures in the following sense: the higher this quantity is, the more delocalized is the density, the higher is the uncertainty and the smaller is the accuracy in predicting the localization of the particle. The sum of these two Shannon entropies is lowerbounded by means of the well-known (Shannon-entropy-based) entropic uncertainty relation conjectured by Everett and Hirschman [141,142] and independently proved by Beckner and Bialynicki-Birula and Mycielski [143,144]

(86) S[ρ]+S[γ]≥D(1+lnπ),

which improves [140,144] the standard Heisenberg relation (31).

These two entropies have been rigorously shown to be upperbounded. Indeed, it was variationally shown for both three [100] and *D*-dimensional [101] systems that the entropy S[ρ] is sharply bounded as

(87) $$S\left[\rho \right]\leq A\left(\alpha ,\beta \right)+\beta \ln\langle r^{\alpha }\rangle +\left(D-\alpha \beta \right)\langle \ln r\rangle ;\;\forall \beta >0,\alpha >-D$$

in terms of the expectation values $\langle r^{\alpha }\rangle$ and $\langle \ln r\rangle$, with

(88) $$A\left(\alpha ,\beta \right)=\beta +\ln\frac{\Omega_{D}\;\Gamma \left(\beta \right)}{\left|\alpha \right|\beta^{\beta }},$$

This inequality with $\beta =\frac{D}{\alpha }$ provides the upper bound (see also [145])

(89) $$S\left[\rho \right]\leq \frac{D}{\alpha }+\ln\left[\frac{2\pi^{\frac{D}{2}}}{\alpha }{\left(\frac{\alpha }{D}\right)}^{\frac{D}{\alpha }}\frac{\Gamma \left(\frac{D}{\alpha }\right)}{\Gamma \left(\frac{D}{2}\right)}\right]+\frac{D}{\alpha }\ln\langle r^{\alpha }\rangle ;\;\forall \alpha >0.$$

Then, for α=2 one has the upper bound

(90) $$S\left[\rho \right]\leq \frac{D}{2}\ln\left(\frac{2\pi e}{D}\langle r^{2}\rangle \right),$$

so that for a given $\langle r^{2}\rangle$ the Shannon entropy is maximum for a Gaussian density of covariance matrix $R=\frac{\langle r^{2}\rangle }{D}I$, where *I* is the identity matrix. Similar upper bounds can be obtained for the momentum entropy S[γ] in terms of $\langle p^{\alpha }\rangle$ and $\langle \ln p\rangle$. Thus, we can correlate the position-momentum entropic uncertainty S[ρ]+S[γ] and the Heisenberg product $\langle r^{\alpha }\rangle \langle p^{\alpha }\rangle$; this gives rise in particular the following relation between the Shannon entropic uncertainty and the familiar Heisenberg uncertainty:

(91) $$S\left[\rho \right]+S\left[\gamma \right]\leq D\ln\left[\frac{2\pi e}{D}{\left(\langle r^{2}\rangle \langle p^{2}\rangle \right)}^{\frac{1}{2}}\right],$$

which emphasizes the uncertainty character of the the Shannon entropic sum and generalizes the corresponding three dimensional result [146].

Similarly, another variational upper bound to S[ρ] and S[γ] can be obtained via logarithmic expectation values; namely,

(92) $$\;S\left[\rho \right]\leq \frac{1}{2}+\ln\left(\sqrt{2\pi }\;\;\Omega_{D}\right)+\ln\Delta \left(\ln r\right)+D\langle \ln r\rangle ,$$

(93) $$S\left[\gamma \right]\leq \frac{1}{2}+\ln\left(\sqrt{2\pi }\;\;\Omega_{D}\right)+\ln\Delta \left(\ln p\right)+D\langle \ln p\rangle .$$

Then, by summing these two expressions and taking into account (41), the relation of the Shannon entropic uncertainty (86) and the logarithmic uncertainty relation follows in a straighforward manner.

For central potentials the previous lower and upper bounds to the Shannon entropies can be improved since the position and momentum densities of the *D*-dimensional state (n,l,{μ}) are given by $\rho_{n,l,\{\mu \}}\left(r\right)$ and $\gamma_{n,l,\{\mu \}}\left(p\right)$. This is because both densities can be decomposed into two radial and angular parts according to Equations (20) and (22), and then the corresponding position and momentum Shannon entropies (84)–(85) factorize as

(94) $$\begin{array}{ccc} S[\rho_{n,l,\{\mu \}}] & = & S\left[R_{n,l}\right]+S\left[Y_{l,\{\mu \}}\right], \end{array}$$

(95) $$\begin{array}{ccc} S[\gamma_{n,l,\{\mu \}}] & = & S\left[M_{n,l}\right]+S\left[Y_{l,\{\mu \}}\right], \end{array}$$

respectively, where the position and momentum radial parts of the entropies are given by

(96) $$\begin{array}{ccc} S(R_{n,l}) & = & -\int r^{D-1}|R_{nl}{\left(r\right)|}^{2}\ln{\left|R_{nl}\left(r\right)\right|}^{2}\;dr, \end{array}$$

(97) $$\begin{array}{ccc} S(M_{n,l}) & = & -\int p^{D-1}|M_{nl}{\left(p\right)|}^{2}\ln{\left|M_{nl}\left(p\right)\right|}^{2}\;dp, \end{array}$$

respectively, and the angular entropy part is given by the entropic functional of the hyperspherical harmonics as

(98) $$\begin{array}{ccc} S\left[Y_{l,\left\{\mu \right\}}\right] & = & -\int_{S_{D-1}}|Y_{l,\left\{\mu \right\}}\left(\Omega_{D-1}\right){|}^{2}\ln{\left|Y_{l,\left\{\mu \right\}}\left(\Omega_{D-1}\right)\right|}^{2}d\Omega_{D-1} \end{array}$$

Note that, contrary to the radial parts $S(R_{n,l})$ and $S(M_{n,l})$ which require the knowledge of the corresponding radial eigenfunctions to go ahead, the angular part $S\left[Y_{l,\left\{\mu \right\}}\right]$ is under control. This is because the entropy of the hyperspherical harmonics [147,148] can be expressed as

(99) $$\begin{array}{ccc} S\left[Y_{l,\left\{\mu \right\}}\right] & = & -B_{1}\left(l,\left\{\mu \right\}\right)+\sum_{j=1}^{D-2}S\left({\tilde{C}}_{\mu_{j}-\mu_{j+1}}^{\left(\alpha_{j}+\mu_{j+1}\right)}\right),\;D\geq 2, \end{array}$$

with the constant

(100) $$\begin{array}{cc} B_{1}\left(l,\left\{\mu \right\}\right) & =\ln2\pi -2\sum_{j=1}^{D-2}\mu_{j+1}\left[\psi (2\alpha_{j}+\mu_{j}+\mu_{j+1}\right.\\ \; & \;\left.-\psi \left(\alpha_{j}+\mu_{j}\right)-\ln2-\frac{1}{2(\alpha_{j}+\mu_{j})}\right], \end{array}$$

where $\psi \left(z\right)=\Gamma^{\prime }\left(z\right)/\Gamma \left(z\right)$ is the digamma function, and $S({\tilde{C}}_{n}^{\left(\lambda \right)})$ denotes the entropy-like functional of the orthonormal Gegenbauer polynomial ${\tilde{C}}_{n}^{\left(\lambda \right)}\left(x\right)$ given by

(101) $$S\left[{\tilde{C}}_{n}^{\left(\lambda \right)}\right]:=-\int_{-1}^{+1}{\left[{\tilde{C}}_{n}^{\left(\lambda \right)}\left(x\right)\right]}^{2}\ln{\left[{\tilde{C}}_{n}^{\left(\lambda \right)}\left(x\right)\right]}^{2}\;{\left(1-x^{2}\right)}^{\lambda -\frac{1}{2}}dx.$$

This entropy can be expressed by means of the values of the quadratic logarithmic potential of Gegenbauer polynomials ${\tilde{C}}_{n}^{\left(\lambda \right)}\left(x\right)$ at the polynomial’s zeros [147,148]. Note that the angular entropy $S[Y_{l,\{\mu \}}]$ does not depend on *n*, and its maximum value is $S\left[Y_{0,\left\{0\right\}}\right]=\ln\Omega_{D}$, which occurs for the *S*-wave states; that is when $\left(l,\left\{\mu \right\}\right)=\left(0,0\right)$. So, it is equal to ln(2π) for D=2, and ln(4π) for D=3. See [148,149] for further specific details about these integral functionals of the Gegenbauer polynomials and the hyperspherical harmonics.

First, to improve the Shannon-entropy-based uncertainty relation (86) for central potentials we use the expressions (21) and (23) of the position and momentum densities into the radial Shannon entropies (96)–(97), obtaining [118]

(102) $$\begin{array}{ccc} S(R_{n,l}) & = & S\left[\omega_{n,l}\right]+\left(D-1\right)\langle \ln r\rangle , \end{array}$$

(103) $$\begin{array}{ccc} S(M_{n,l}) & = & S\left[{\tilde{\omega }}_{n,l}\right]+\left(D-1\right)\langle \ln p\rangle , \end{array}$$

where S[ω] denotes the Shannon entropy of the one-dimensional density $\omega_{n,l}\left(r\right)={\left|u_{n,l}\left(r\right)\right|}^{2}$:

$$S\left[\omega_{n,l}\right]=-\int_{0}^{\infty }\omega_{n,l}\left(r\right)\ln\omega_{n,l}\left(r\right)dr.$$

and the logarithmic expectation value 〈lnr〉 has the value

$$\langle \ln r\rangle =\int_{R^{D}}\rho_{n,l,\{\mu \}}\left(\vec{r}\right)\ln r\;d^{D}r=\int_{0}^{\infty }\omega_{n,l}\left(r\right)\ln r\;dr$$

The momentum quantities $S[{\tilde{\omega }}_{n,l}]$ and 〈lnp〉 of the reduced density ${\tilde{\omega }}_{n,l}\left(p\right)={\left|{\tilde{u}}_{n,l}\left(p\right)\right|}^{2}$ are correspondingly defined. Then, keeping in mind the expressions (94), (95), (102) and (103), we have the total position-momentum Shannon entropy

(104) $$S\left[\rho_{n,l,\{\mu \}}\right]+S\left[\gamma_{n,l,\{\mu \}}\right]=S\left[\omega_{n,l}\right]+S\left[{\tilde{\omega }}_{n,l}\right]+\left(D-1\right)\left(\langle \ln r\rangle +\langle \ln p\rangle \right)+2\;S\left(Y_{l,\{\mu \}}\right),$$

where the only terms which depend on the (unknown) analytical form of the potential $V_{D}\left(r\right)$ are the sums $S\left[\omega \right]+S\left[\tilde{\omega }\right]$ and 〈lnr〉+〈lnp〉. However, Rudnicki et al. [118] have found by use of a limiting case of the De-Carli Hankel-transform integral inequality that the Shannon reduced sum is lowerbounded as

(105) $$S\left[\omega \right]+S\left[\tilde{\omega }\right]\geq 2l+D+2\ln\left[\frac{\Gamma \left(l+\frac{D}{2}\right)}{2}\right]-\left(2l+D-1\right)\psi \left(l+\frac{D}{2}\right)$$

Thus, from this inequality and the previous central-potential logarithmic uncertainty relation (63) one finally has the **Shannon-entropy-based uncertainty relation for any central potential**:

(106) $$S\left[\rho_{n,l,\{\mu \}}\right]+S\left[\gamma_{n,l,\{\mu \}}\right]\geq B_{l,\{\mu \}},$$

with the constant

(107) $$\begin{array}{ccc} B_{l,\{\mu \}} & = & 2l+D+2\ln\left[\frac{\Gamma \left(l+\frac{D}{2}\right)}{2}\right]-\left(2l+D-1\right)\psi \left(l+\frac{D}{2}\right)\\ & & +\left(D-1\right)\left(\psi \left(\frac{2l+D}{4}\right)+\ln2\right)+2\;S\left(Y_{l,\{\mu \}}\right) \end{array}$$

where $S(Y_{l,\{\mu \}})$ is given by Equation (98)–(99). This lower bound depends on the angular hyperquantum numbers (l,{μ}) and the dimensionality *D*, but not on the principal quantum number *n*. The latter is because the radial wavefunction is unknown. The comparison of the general bound D(1+lnπ) given by (86) and the central bound $B_{l,\{\mu \}}$ shows that the larger the value of *l*, the bigger is the new central bound, and the larger the improvement with respect to the general bound. Moreover, for D=3 the central bound is bigger (so, better) than the general one for all values (l,m) except for l=m=0. For D≠3 the bound might not be improved for more than one set of values (l,{μ}), such as for example the case of the values $\left(l,\mu_{2},\mu_{3}\right)=\left(0,0,0\right)$, (1,0,0) and (1,1,0), when D=4. The latter phenomenon is an open problem for the future; it might be due either to the separation (104) between the sum of the Shannon entropies of the reduced densities and the logarithmic uncertainty sum, and/or to the fact that, when l=0, the logarithmic uncertainty relation (63) is not sharp enough in this case. See [118] for further details.

To improve the entropic upper bounds (89) and (90) for central potentials we can use the Rényi maximization approach of Costa et al. [150] with a covariance constraint. This has been illustrated by Sánchez-Moreno et al. [151] to improve the upper bound (90) in terms of the expectation value $\langle r^{2}\rangle$, obtaining the sharp upper bound

(108) $$S\left[\rho \right]\leq \frac{D}{2}\ln\left(\frac{2\pi e}{D}\langle r^{2}\rangle \right)+L\left(\Omega_{D-1}\right)$$

where $L(\Omega_{D-1})$, which represents the loss of entropy due to the angular part of the state’s wavefunction (i.e., it is coming from the hyperpherical harmonics), is given by

(109) $$L\left(\Omega_{D-1}\right)=\frac{1}{2}\sum_{k=1}^{D-2}\left(\left(D-k\right)\ln\langle \sin^{2}\theta_{k}\rangle +\ln\langle \cos^{2}\theta_{k}\rangle \right)-\ln2+\frac{D}{2}\ln D,$$

and

(110) $$\langle \cos^{2}\theta_{k}\rangle =\frac{2\mu_{k}\left(\mu_{k}+D-k-1\right)-2\mu_{k+1}\left(\mu_{k+1}+D-k-2\right)+D-k-3}{4\mu_{k}\left(\mu_{k}+D-k-1\right)+\left(D-k+1\right)\left(D-k-3\right)}.$$

and, of course, $\langle \sin^{2}\theta_{k}\rangle =1-\langle \cos^{2}\theta_{k}\rangle$. From the last two expressions we observe that there are some special configurations for which the loss of entropy $L(\Omega_{D-1})$ vanishes; namely when

(111) $$L\left(\Omega_{D-1}\right)=0\Leftrightarrow \left\{\begin{array}{c} \mu_{i}=0,\;\forall i\;\mathrm{for}(nS)-\mathrm{states}\;\\ m\equiv \mu_{D-1}=\pm \;2,\;\;\mathrm{and}\;\;\mu_{i}=D-i+1,\;\;\forall i<D-1 \end{array}\right.$$

Note that the first configuration corresponds to the (nS) states; they have spherically symmetric wavefunctions since the angular part (the hyperspherical harmonics) is a constant and thus there is no loss of entropy. The second configuration is the interesting one because the wave function is not spherically symmetric since the magnetic number *m* is ±2 and the remaining angular hyperquantum numbers $\mu_{i}$ of the state have special values. The phenomenon of the vanishing entropy loss in this special non-spherically-symmetric configuration of angular numbers is an interesting physical problem, not yet fully understood. Note that for three-dimensional central systems, the previous upper bound simplifies as

(112) $$S\left[\rho \right]\leq \frac{3}{2}\ln\left(\frac{2\pi e}{3}\langle r^{2}\rangle \right)+L\left(\Omega_{2}\right),$$

where the loss of entropy $L(\Omega_{2})$ is

(113) $$L\left(\Omega_{2}\right)=\frac{1}{2}\ln\left(\frac{2l\left(l+1\right)-2m^{2}-1}{4l(l+1)-3}\right)+\ln\left(\frac{l\left(l+1\right)+m^{2}-1}{4l(l+1)-3}\right)+\frac{3}{2}\ln3,$$

since the involved trigonometric expectation values are

$$\langle \cos^{2}\theta \rangle =\frac{2l\left(l+1\right)-2m^{2}-1}{4l(l+1)-3},\;\langle \sin^{2}\theta \rangle =\frac{2l\left(l+1\right)+2m^{2}-2}{4l(l+1)-3}$$

Note that $L(\Omega_{2})=0$ if and only if l=m=0 (nS-states) or l=3 and m=±2. To check the accuracy of the bound (90) and its improvement (112) for central potentials, the mutual comparison of these bounds and the exact position entropy for various bound states of two relevant classes of central potentials has been analytically and numerically examined in detail [62]: the *D*-dimensional and three-dimensional hydrogenic and oscillator-like systems. It has been found for these three dimensional cases that the general bound (90) and the central bound (112) for l=m=0 and l=3, m=2 have the same values, what confirms the theoretical observation (111). Moreover, for the circular states (l=m) the improvement of the central bound is remarkable, when the spherical harmonics are concentrated around the equatorial region with θ near π/2. The size of the improvement is specially relevant for m=0 when the spherical harmonics are concentrated around the polar regions with θ near 0 and π.

Note that the entropic uncertainty relation (106) and the upper bounds (112) can be further improved for all the stationary states of large, specific classes of central potentials, such as e.g., the power-law potentials, but this is still an open issue up until now. However, for highly-excited states of one-dimensional power-law potentials of the type $x^{2k}$ with k∈N and x∈R the asymptotics of the position and momentum Shannon entropies have been determined [129] by means of the WKB approximation. We have found that for highly excited states both position and momentum entropies have a logarithmic dependence on its quantum number not only for both harmonic (k=1) and anharmonic (k≠1) oscillators.

### 4.3. The Rényi Entropies

Here, we consider the position and momentum properties of the Rényi entropies of a multidimensional quantum state. We show their universally-valid lower and upper bounds by means of the position and momentum Heisenberg-like and logarithmic measures. Then, the uncertainty character for the sum of the position and momentum Rényi entropies is manifested. Later, the Rényi entropies for the quantum states of arbitrary central potentials are examined. First we heuristically argue how the spherically-symmetric effects of the quantum potential improve the associated position-momentum uncertainty relation. Second, we give the rigorous upper bounds of the Rényi entropies for central potentials.

The Rényi entropies for the position probability density ρ(r) are defined [27,28] by

(114) $$R_{q}\left[\rho \right]=\frac{1}{1-q}\ln\int_{R_{D}}{\left[\rho \left(r\right)\right]}^{q}\;dr,\;0<q<\infty ,\;\;q\neq 1,$$

They supply a family of entropic measures of quantum states, depending on a real parameter *q*. The order parameter *q* allows to vary, by increasing or decreasing its value, the contribution of the probability density over different regions. The higher the value of *q* the more concentrated is the function ${\left[\rho \left(r\right)\right]}^{q}$ around the local maxima of the distribution, while the lower values have the effect of smoothing that function over its whole definition region. The Rényi entropies provide various complementary ways to quantify the extent of ρ(r) all over the hyperspace, including numerous relevant quantities as special cases, such as e.g., the disequilibrium $D\left[\rho \right]=\exp(-R_{2}\left[\rho \right])$ and the (previously considered) Shannon entropy $S\left[\rho \right]=\lim_{p\to 1}R_{p}\left[\rho \right]$. The corresponding quantities $R_{q}\left[\gamma \right]$ for the momentum density γ(p) are given by

(115) $$R_{q^{*}}\left[\gamma \right]=\frac{1}{1-q^{*}}\ln\int_{R_{D}}{\left[\gamma \left(p\right)\right]}^{q^{*}}\;dp,\;0<q^{*}<\infty ,\;\;q^{*}\neq 1,$$

Beyond the monotonicity relations [44] given by

(116) $$R_{p}\left[\rho \right]\geq R_{q}\left[\rho \right],\;\mathrm{if}\;p\leq q;\;\mathrm{and}\;\frac{p-1}{p}R_{p}\left[\rho \right]\geq \frac{q-1}{q}R_{q}\left[\rho \right],\;\mathrm{if}\;p\geq q>1$$

(which allows one to lowerbound all the Rényi entropies by means of the second-order entropy as $R_{q}\left[\rho \right]\geq \frac{1}{2}R_{2}\left[\rho \right],\;\mathrm{for}\;q>0$), the most important property of these quantities is the Rényi-entropy-based uncertainty relation given by

(117) $$R_{q}\left[\rho_{\left\{n_{i}\right\}}\right]+R_{q^{*}}\left[\gamma_{\left\{n_{i}\right\}}\right]\geq D\ln\left(\pi q^{\frac{1}{2q-2}}{q^{*}}^{\frac{1}{2q^{*}-2}}\right)$$

of general validity for *D*-dimensional quantum systems which was proved by Zozor, Portesi and Vignat [46] for arbitrary indices, extending the one-dimensional relation previously found by Bialynicki-Birula [47] and Zozor and Vignat [152] for conjugated indices (i.e., when $\frac{1}{q}+\frac{1}{q^{*}}=2$). See [42] for a review. Therein, we can learn that these uncertainty relations are saturated by the Gaussian distributions.

Moreover, the position and momentum Rényi entropies can be upperbounded. Indeed the maxent problem can provide variational upper bounds to the Rényi entropy $R_{q}\left[\rho \right]$ given by (114). It has been found, in particular, that the upper bounds

(118) $$R_{q}\left[\rho \right]\leq \frac{1}{1-q}\ln\left\{L_{1}\left(q,k,D\right){\langle r^{k}\rangle }^{-\frac{D}{k}\left(q-1\right)}\right\},\;q>1,\;k=1,2,...$$

and

(119) $$R_{q}\left[\rho \right]\leq \frac{1}{1-q}\ln\left\{L_{2}\left(q,k,D\right){\langle r^{-k}\rangle }^{-\frac{D}{k}\left(q-1\right)}\right\},\;q>1$$

with k=1,2,⋯ so that $k<\frac{D}{q}\left(q-1\right)$. The functions $L_{i}\left(q,k,D\right)$, i=1 and 2, are known to have the expressions

(120) $$L_{1}\left(q,k,D\right)=\frac{qk}{D(q-1)+kq}{\left\{\frac{k\Gamma \left(D/2\right){\left[\frac{D(q-1)}{D(q-1)+kq}\right]}^{\frac{D}{k}}}{2\pi^{\frac{D}{2}}B\left(\frac{q}{q-1},\frac{D}{k}\right)}\right\}}^{q-1}$$

and

(121) $$L_{2}\left(q,k,D\right)=\frac{qk}{D(q-1)-kq}{\left\{\frac{k\Gamma \left(D/2\right){\left[\frac{D(q-1)-kq}{D(q-1)}\right]}^{\frac{D}{k}}}{2\pi^{\frac{D}{2}}B\left(\frac{D}{k}-\frac{1}{q-1},\frac{q}{q-1}\right)}\right\}}^{q-1}$$

where B(x,y)=Γ(x)Γ(y)/Γ(x+y) is Euler’s beta function. We have used the variational bounds [153,154] to the entropic moments $W_{q}\left[\rho \right]=\int_{R_{D}}{\left[\rho \left(r\right)\right]}^{q}\;dr$ with a single radial expectation value as constraint. These bounds (118)–(121) extend and generalize (see also [145]) similar bounds obtained in the one-dimensional [155,156] and three-dimensional [157] cases used in various contexts, ranging from finances to atomic physics. In momentum space similar expressions to (118)–(121) can be variationally derived for the corresponding entropies and constraints. In particular, for k=2 one has [151] the upper bound

(122) $$R_{q}\left[\rho \right]\leq B_{D}\left(q\right)+\frac{D}{2}\ln\left(\frac{\langle r^{2}\rangle }{D}\right),$$

with

(123) $$B_{D}\left(q\right)=\left\{\begin{array}{ccc} \frac{D}{2}\ln\left(\frac{\pi ((2+D)q-D)}{q-1}\right)+\frac{1}{q-1}\ln\left(\frac{(2+D)q-D}{2q}\right)+\ln\left(\frac{\Gamma \left(\frac{q}{q-1}\right)}{\Gamma \left(\frac{(2+D)q-D}{2(q-1)}\right)}\right) & \mathrm{if} & q>1,\;\\ \frac{D}{2}\ln\left(\frac{\pi ((2+D)q-D)}{1-q}\right)-\frac{q}{1-q}\ln\left(\frac{(2+D)q-D}{2q}\right)-\ln\left(\frac{\Gamma \left(\frac{q}{1-q}\right)}{\Gamma \left(\frac{(2+D)q-D}{2(1-q)}\right)}\right) & \mathrm{if} & q\in \left(\frac{D}{D+2},1\right),\; \end{array}\right.$$

to the position Rényi entropy, and

(124) $$R_{q^{*}}\left[\gamma \right]\leq B_{D}\left(q^{*}\right)+\frac{D}{2}\ln\left(\frac{\langle p^{2}\rangle }{D}\right),$$

to the momentum Rényi entropy. Thus, we can obtain in particular that the position-momentum Rényi entropy sum and the position-momentum Heisenberg uncertainty $\langle r^{2}\rangle \langle p^{2}\rangle$ are related as

(125) $$R_{q}\left[\rho \right]+R_{q^{*}}\left[\gamma \right]\leq B_{D}\left(q\right)+B_{D}\left(q^{*}\right)-D\ln D+\frac{D}{2}\left(\langle r^{2}\rangle \langle p^{2}\rangle \right),$$

which emphasizes the uncertainty character of the position-momentum Rényi entropy sum and generalizes the corresponding three dimensional result [146] to *D* dimensions.

**For central potentials**, the Rényi entropies have not yet been explicitly obtained by means of the state’s hyperquantum numbers for reasons similar to the Shannon case. Indeed, in this case the Rényi entropies for an arbitrary *D*-dimensional state characterized by the position and momentum probability densities $\rho_{n,l,\{\mu \}}\left(r\right)$ and $\gamma_{n,l,\{\mu \}}\left(p\right)$ are given, according to Equations (21) and (23), by

(126) $$R_{q}\left[\rho_{n,l,\{\mu \}}\right]=R_{q}\left[u_{n,l}\right]+R_{q}\left[Y_{l,\{\mu \}}\right],$$

(127) $$R_{q*}\left[\gamma_{n,l,\{\mu \}}\right]=R_{q*}\left[{\tilde{u}}_{n,l}\right]+R_{q*}\left[Y_{l,\{\mu \}}\right],$$

where the symbols $R_{q}\left[u_{n,l}\right]$, $R_{q*}\left[{\tilde{u}}_{n,l}\right]$ and $R_{q}\left[Y_{l,\{\mu \}}\right]$ denote the position and momentum radial and angular Rényi entropies for *D*-dimensional quantum state (n,l,{μ}), respectively. The radial Rényi entropies are given by

(128) $$\begin{array}{ccc} R_{q}\left[u_{n,l}\right] & = & \frac{1}{1-q}\ln\int_{0}^{\infty }dr\;r^{\left(D-1)(1-q\right)}{\left|u_{n,l}\left(r\right)\right|}^{2q} \end{array}$$

(129) $$\begin{array}{ccc} R_{q*}\left[{\tilde{u}}_{n,l}\right] & = & \frac{1}{1-q*}\ln\int_{0}^{\infty }dr\;r^{\left(D-1)(1-q*\right)}{\left|u_{n,l}\left(r\right)\right|}^{2q*} \end{array}$$

in position and momentum spaces, respectively, and the angular Rényi entropies are given by

(130) $$R_{q}\left[Y_{l,\{\mu \}}\right]:=\frac{1}{1-q}\ln\Lambda_{q}\left[Y_{l,\{\mu \}}\right].$$

with the entropic moments of the hyperspherical harmonics [158]

(131) $$\begin{array}{ccc} \Lambda_{q}\left[Y_{l,\{\mu \}}\right] & = & \int_{S_{D-1}}{\left|Y_{l,\{\mu \}}\left(\Omega_{D-1}\right)\right|}^{2q}\;d\Omega_{D-1}\\ & = & 2\pi N_{l,\{\mu \}}^{2q}\prod_{j=1}^{D-2}\int_{0}^{\pi }{\left[C_{\mu_{j}-\mu_{j+1}}^{\left(\alpha_{j}+\mu_{j+1}\right)}\left(\cos\theta_{j}\right)\right]}^{2q}{\left(\sin\theta_{j}\right)}^{2q\mu_{j+1}+2\alpha_{j}}\;d\theta_{j}, \end{array}$$

where the normalization constant $N_{l,\{\mu \}}$ is given by

(132) $$N_{l,\{\mu \}}^{2}=\frac{1}{2\pi }\prod_{j=1}^{D-2}\frac{\left(\alpha_{j}+\mu_{j}\right)\left(\mu_{j}-\mu_{j+1}\right)!{\left[\Gamma \left(\alpha_{j}+\mu_{j+1}\right)\right]}^{2}}{\pi \;2^{1-2\alpha_{j}-2\mu_{j+1}}\Gamma \left(2\alpha_{j}+\mu_{j}+\mu_{j+1}\right)}.$$

Note that, according Equations (7) and (130)–(132), the hyperspherical harmonics $Y_{l,\{\mu \}}\left(\Omega_{D-1}\right)$ and consequently the angular Rényi entropies $R_{q}\left[Y_{l,\{\mu \}}\right]$ do not depend on the principal hyperquantum number *n* but they do depend on the the angular hyperquantum numbers and the dimensionality *D*. Moreover, the integral functionals involved in (131) are the Rényi-like functionals of the Gegenbauer polynomials, which are under control since they can be analytically calculated by two recent methodologies: one based on the Srivastava’s linearization method [159] and another one based on the combinatorial Bell polynomials [160].

**To improve the Rényi-entropy-based uncertainty relation (117) for central potentials** we take into account the expressions (21) and (23) of the position and momentum densities, respectively, so that the sum of the position and momentum Rényi entropies can be expressed as

(133) $$R_{q}\left[\rho_{n,l,\{\mu \}}\right]+R_{q*}\left[\gamma_{n,l,\{\mu \}}\right]=R_{q}\left[u_{n,l}\right]+R_{q*}\left[{\tilde{u}}_{n,l}\right]+R_{q}\left[Y_{l,\{\mu \}}\right]+R_{q*}\left[Y_{l,\{\mu \}}\right]$$

Recently, it has been heuristically found that

(134) $$R_{q}\left[u_{n,l}\right]+R_{q*}\left[{\tilde{u}}_{n,l}\right]\geq \frac{2q\ln A(2q)}{q-1}+\frac{2q^{*}\ln A\left(2q^{*}\right)}{q^{*}-1},$$

(whose validity conditions have not yet been established) with the constant

$$A\left(q\right)=2^{\frac{2-D}{2q}}\frac{q^{\frac{1}{2}\left(\frac{1}{2}+l+\frac{D}{2}-1+\frac{D-1}{2}\frac{2-q}{q}+\frac{1}{q}\right)}}{\Gamma {\left(\left(l+\frac{D}{2}-1+\frac{D-1}{2}\frac{2-q}{q}+\frac{1}{2}\right)\frac{q}{2}+\frac{1}{2}\right)}^{\frac{1}{q}}}.$$

Then, by combining the last three expressions we finally have the Rényi-entropy-based position-momentum uncertainty relation for quantum systems with a central potential as

(135) $$R_{q}\left[\rho_{n,l,\{\mu \}}\right]+R_{q*}\left[\gamma_{n,l,\{\mu \}}\right]\geq \frac{2q\ln A(2q)}{q-1}+\frac{2q^{*}\ln A\left(2q^{*}\right)}{q^{*}-1}+R_{q}\left[Y_{l,\{\mu \}}\right]+R_{q*}\left[Y_{l,\{\mu \}}\right]$$

This heuristic uncertainty relation gives a lower bound for the sum of the position and momentum Rényi entropies by means of the angular hyperquantum numbers (l,{μ}). Despite this relation does not depend on the analytical form of the central potential, it is not valid for all central potentials for the reason mentioned above. However, it has been numerically shown to be fulfilled by various large classes of qualitatively different central potentials such as e.g., the oscillator and hydrogenic-like potentials. Moreover, in the limits $(q\to 1,q^{*}\to 1$) one realizes that the uncertainty inequality (135) simplifies as

(136) $$\begin{array}{ccc} S\left[\rho_{n,l,\{\mu \}}\right]+S\left[\gamma_{n,l,\{\mu \}}\right] & = & S\left(u_{n,l}\right)+S\left({\tilde{u}}_{n,l}\right)+2S\left(Y_{l,\{\mu \}}\right)\\ & \geq & 2l+D+2\ln\frac{\Gamma \left(l+\frac{D}{2}\right)}{2}-2l\psi \left(l+\frac{D}{2}\right)+2S\left(Y_{l,\{\mu \}}\right), \end{array}$$

which improves the rigorous position-momentum Shannon-entropy-based uncertainty relation for central potentials given by (107). The lower bound (136) is always larger or equal to the general lower bound (86); so, even when l=0, where the latter bound failed because a logarithmic uncertainty-like inequality used for its derivation is not sharp enough. Nevertheless, the rigorous proof of the heuristic position-momentum Rényi-entropy based uncertainty relation (135) or, at least its validity conditions, remains an open problem for the future.

**To improve the entropic upper bounds (122)–(124) for central potentials** the Rényi maximization procedure of Costa et al. [150] can be used again to find [151] the following sharp upper bound in terms of the expectation value $\langle r^{2}\rangle$:

(137) $$R_{q}\left[\rho \right]\leq B_{D}\left(q\right)+\frac{D}{2}\ln\left(\frac{\langle r^{2}\rangle }{D}\right)+L\left(\Omega_{D-1}\right),$$

where the spherical-symmetry entropy effects of the potential are contained in the quantity $L(\Omega_{D-1})$, which represents the loss of entropy due to the angular part of the state’s wavefunction, is given by Equation (109). For the three-dimensional quantum systems with a central potential, this expression boils down as

(138) $$R_{q}\left[\rho \right]\leq B_{3}\left(q\right)+\frac{3}{2}\ln\left(\frac{\langle r^{2}\rangle }{3}\right)+L\left(\Omega_{2}\right),$$

where $L(\Omega_{2})$ is given by the expression (113) which only depends on the quantum numbers (l,m) which control the spherical harmonics $Y_{l,m}$ involved in the three dimensional wavefunction of the system. Then, the inequalities (137) and (138) provide the improvement to the general position Rényi-entropy upper bounds given by (122) because of the spherical symmetry of the potential for the *D* and two-dimensional quantum systems, respectively. It has been found [62] that the general and central position upper bounds coincide for l=m=0 and l=3,m=2 in accordance to the theoretical result (111). The improvement of the bound, as given by the loss of entropy $L(\Omega_{2})$ is independent of the specific form of the potential. Thus, the best improvement occurs for the cases l=m and m=0.

Similar improvements follow for the momentum upper bounds (124) in terms of the second-order radial expectation value $\langle p^{2}\rangle$, so that we have the sharp inequality

(139) $$R_{q^{*}}\left[\gamma \right]\leq B_{D}\left(q^{*}\right)+\frac{D}{2}\ln\left(\frac{\langle p^{2}\rangle }{D}\right)+L\left(\Omega_{D-1}\right)$$

Then, the position-momentum Rényi entropy sum and the position-momentum Heisenberg uncertainty $\langle r^{2}\rangle \langle p^{2}\rangle$ are related as

(140) $$R_{q}\left[\rho \right]+R_{q^{*}}\left[\gamma \right]\leq B_{D}\left(q\right)+B_{D}\left(q^{*}\right)-D\ln D+\frac{D}{2}\left(\langle r^{2}\rangle \langle p^{2}\rangle \right)+2\;L\left(\Omega_{D-1}\right)$$

for central potentials with dimensionality D≥3.

## 5. Spin Effects on the Heisenberg and Entropic Uncertainty Relations of Multidimensional Quantum Systems

Now we examine the present knowledge of the spin effects on the general and central uncertainty relations of Heisenberg and entropic types for single-fermion systems with spin *s*. Particularly, by use of the Daubechies–Thakkar and Lieb–Thirring inequalities, we show the spin effects on the Heisenberg uncertainty relations given by expressions (32) and (60), and on the Fisher-information-based uncertainty relations given by expressions (81)–(83). It remains open the investigation of the spin effects of uncertainty inequalities of Shannon (see (86), (91), (106), (136)) and Rényi (see (117), (125), (135)) types.

To obtain the **spin-dependent Heisenberg uncertainty relations** we begin with the Daubechies–Thakkar uncertainty inequality [67,95,161] of the momentum radial expectation value of order *k* and the position entropic moment of order $1+\frac{k}{D}$:

(141) $$\langle p^{k}\rangle \geq K_{D}\left(k\right)\;q^{-\frac{k}{d}}\int_{R_{D}}{\left[\rho \left(\vec{r}\right)\right]}^{1+\frac{k}{D}}\;d^{D}\vec{r},$$

where k>0, q=2s+1 is the number of spin states, and

(142) $$K_{D}\left(k\right)=\frac{D}{k+D}{\left(2\pi \right)}^{k}\frac{{\left[\Gamma \left(1+\frac{D}{2}\right)\right]}^{k/D}}{\pi^{k/2}}\;.$$

Lieb (see e.g., [162]) previously conjectured the case k=2 in Equation (141); later, weaker versions of it were rigorously proved [163]. Moreover, Daubechies [161] rigorously proved this inequality with constant $K_{D}^{\prime }\left(k\right)=K_{D}\left(k\right)\times B\left(D,k\right)$ wnere $B\left(D,k\right)={\left\{\Gamma \left(\frac{D}{k}\right)\inf_{a>0}\left[a^{-\frac{D}{k}}{\left(\int_{a}^{\infty }du\;e^{-u}\left(u-a\right)u^{-1}\right)}^{-1}\right]\right\}}^{-\frac{k}{D}}$. Other authors have published some rigorous *D*-dimensional bounds of the same type [164,165] with much less accuracy.

Then, the combination of the lower bound (141) to the momentum expectation value $\langle p^{k}\rangle$ and the variational bounds (118) to the involved position entropic moments [153,154] allows us to find [67,166] the following spin-dependent Heisenberg-like uncertainty relations for single-fermion systems:

(143) $${\langle r^{\alpha }\rangle }^{\frac{k}{\alpha }}\langle p^{k}\rangle \geq F\left(D,\alpha ,k\right)\;q^{-\frac{k}{D}},$$

with

(144) $$\begin{array}{ccc} F(D,\alpha ,k) & = & K_{D}\left(k\right)\times \frac{{\left(1+\frac{k}{D}\right)}^{1+\frac{k}{D}}\alpha^{1+\frac{2k}{D}}}{{\left[\Omega_{D}B\left(\frac{D}{\alpha },2+\frac{D}{k}\right)\right]}^{\frac{k}{D}}}\times {\left\{\frac{k^{k}}{{\left[\left(1+\frac{k}{D}\right)\alpha +k\right]}^{\left(1+\frac{k}{D}\right)\alpha +k}}\right\}}^{\frac{1}{\alpha }}, \end{array}$$

where $\Omega_{D}=\frac{2\pi^{D/2}}{\Gamma (D/2)}$ is the volume of the unit hypersphere. This uncertainty relation, which holds for any single-fermion systems with space dimensionality *D* and spin dimensionality q=2s+1, was previously found for k=2 by means of the Lieb–Thirring inequality [166]. Then, for α=k=2, one has that

(145) $$\langle r^{2}\rangle \langle p^{2}\rangle \geq {\left\{\frac{D}{D+1}{\left[\Gamma (D+1)\right]}^{\frac{1}{D}}\right\}}^{2}\;{\left(2s+1\right)}^{-\frac{2}{D}},$$

This generalized (space-spin) uncertainty relation shows a delicate balance of the space and spin dimensionality effects to the position-momentum Heisenberg uncertainty, so that the bound (145) is better (worse) than the spinless bound $D^{2}/4=0.25\;D^{2}$ when *D* is small (large), respectively. Indeed, note that the bound (145) increases when the space dimensionality is increasing, thus the uncertainty relation gets improved; and for large values of *D*, the bound (145) behaves as $D^{2}/e^{2}=0.1353\;D^{2}$. Let us also highlight that for a given *D*-dimensional single-fermion system, the uncertainty relation is less accurate when the spin dimensionality of the system increases. Moreover, one trivially obtains the spin-dependent Heisenberg uncertainty relation for all three-dimensional single-fermion systems

(146) $$\langle r^{2}\rangle \langle p^{2}\rangle \geq {\left(\frac{3}{4}6^{1/3}\right)}^{2}{\left(2s+1\right)}^{-2/3}$$

where the equality is reached for the harmonic oscillator [167]. Then, the spin-dependent Heisenberg uncertainty for electronic systems (s=1/2) is $\langle r^{2}\rangle \langle p^{2}\rangle \geq 1.85733\times 2^{-2/3}=1.17005$.

In addition, uncertainty relations similar to Equation (145), at times with better accuracy, have been recently found [168] by means of a variational procedure based on the extremization of various information-theoretical measures which allows us to determine an extremum-entropy or least-biased distribution compatible with the known data. Indeed, the combination of the Daubechies–Thakkar momentum lower bound (141) with the position entropic moments of the maximizer solution $\rho_{S}\left(r\right)$ of the Shannon MaxEnt problem with the constraints ($\langle r^{0}\rangle =1,\langle r^{\alpha }\rangle$) has allowed us to find

(147) $${\langle r^{\alpha }\rangle }^{\frac{k}{\alpha }}\langle p^{k}\rangle \geq 2^{\frac{(D-2)k}{D}}D^{\frac{\left(\alpha +D)(D+k\right)}{\alpha D}}{\left(D+k\right)}^{-\frac{\alpha +D}{\alpha }}\alpha^{k\left(\frac{1}{D}-\frac{1}{\alpha }\right)}\Gamma {\left(\frac{D}{2}\right)}^{\frac{D+k}{D}}\Gamma {\left(\frac{D}{\alpha }\right)}^{-\frac{k}{D}}\;{\left(2s+1\right)}^{-\frac{k}{D}},$$

with α>0, k>0. Then, one has for three-dimensional single-electron systems (d=3 and q=2) the following uncertainty-like relation

(148) $${\langle r^{\alpha }\rangle }^{\frac{k}{\alpha }}\langle p^{k}\rangle \geq \frac{2^{-\frac{2k}{3}}\pi^{\frac{k}{3}}3^{\frac{\left(\alpha +3)(k+3\right)}{3\alpha }}\Gamma {\left(\frac{3}{\alpha }\right)}^{-\frac{k}{3}}\alpha^{k\frac{\left(\alpha -3\right)}{3\alpha }}}{{\left(k+3\right)}^{1+\frac{3}{\alpha }}},$$

which for the particular case α=2 and k=2, gives

(149) $$\langle r^{2}\rangle \langle p^{2}\rangle \geq \frac{81\sqrt[{6}]{3}\sqrt[{3}]{\pi }}{50\sqrt{5}}=1.30107$$

which clearly improves the previous bound 1.17005. Finally, similar bounds obtained with this Shannon MaxEnt procedure [169,170,171,172] and with extremization methods associated to other information-theoretical measures (Fisher information, Tsallis entropies [173,174]) [168] have been found by various authors for a large number of atoms and molecules.

To obtain the **spin-dependent Fisher-information-based uncertainty relations** we use the inequalities (81) and (145), giving rise to

(150) $$F\left[\rho_{n,l,\{\mu \}}\right]\times F\left[\gamma_{n,l,\{\mu \}}\right]\geq 16{\left[1-\frac{2|m|}{2l+D-2}\right]}^{2}\;{\left\{\frac{D}{D+1}{\left[\Gamma \left(D+1\right)\right]}^{\frac{1}{D}}\right\}}^{2}\;\;{\left(2s+1\right)}^{-2/D}$$

This uncertainty relation holds for all *D*-dimensional single-fermion systems subject to an arbitrary central potential. Let us highlight that for the values $l\equiv \mu_{1}=\ldots =m=0$, one has

(151) $$F\left[\rho_{n,0,\{0\}}\right]\times F\left[\gamma_{n,0,\{0\}}\right]\geq {\left\{\frac{4D}{D+1}{\left[\Gamma (D+1)\right]}^{\frac{1}{D}}\right\}}^{2}{\left(2s+1\right)}^{-\frac{2}{D}},$$

which is the spin-modified expression for the general Fisher-information-based uncertainty relation $F\left[\rho \right]\times F\left[\gamma \right]\geq 4D^{2}$ already mentioned [62]. Note that the bound (151) behaves as $16e^{-2}D^{2}=2.16536\;D^{2}$ for large *D*. Then, here again, it is manifest the delicate balance of the space and spin dimensionality effects which makes the lower bound (151) to be better (worse) than the spinless bound when *D* is small (large). In addition, we observe that the lower bound to the position-momentum Fisher-information-based uncertainty increases when the space dimensionality is increasing; and it decreases when the spin dimensionality is increasing, so that the spin effects worse the uncertainty relation, especially when the space dimensionality decreases. Finally, for the standard (D=3) systems we obtain the uncertainty relation

$$F\left[\rho \right]\times F\left[\gamma \right]\geq 9\times 6^{2/3}\;{\left(2s+1\right)}^{-2/3}$$

for all three-dimensional fermionic systems. So, for electronic systems ($s=\frac{1}{2}$), one has $F\left[\rho \right]\times F\left[\gamma \right]\geq 3^{8/3}=2.1810$.

## 6. Conclusions and Open Problems

Spherical symmetry is one of the most frequent and useful approximations to simplify and solve the Schrödinger equation of quantum systems. In higher dimensions, this approximation often provides a deeper quantitative insight into the quantum structure and dynamics of three-dimensional systems, and in many cases allows for the conceptual understanding of physics in a transparent and intuitive way. Moreover, the solutions of the wave equations of complex physical systems within this approximation are very valuable tools for checking and improving complicated numerical methods used.

Here, we have investigated and reviewed the spatial extension of the D-dimensional stationary states of arbitrary central potentials by means of various spreading quantities of global (radial and logarithmic expectation values, Rényi and Shannon entropies) and local (Fisher information) type. Attention has been focussed on the improvements of the Heisenberg-like and the information-theoretical measures of the associated position and momentum densities because of the spherical symmetry effects of the multidimensional potential and the spin effects of the fermionic system under consideration.

The results have been shown in the form of inequality-based relations because the analytical form of the central potential is not known. In particular, the spherical-symmetry effects on the upper bounds of the Heisenberg-like (radial expectation values) and entropy-like (Fisher, Shannon, Rényi) spreading measures and on the associated uncertainty relations have been examined and discussed, showing their explicit dependence on the angular hyperquantum numbers. In addition, the delicate balance of the space and spin dimensionality effects on the mathematical formulations of the position-momentum uncertainty principle based on the Heisenberg-like measures and the Fisher information has been investigated and discussed.

Let us now mention a few open issues which have been identified. First, the improvement of the Heisenberg-like and entropy-like uncertainty relations for various broad relevant classes of potentials such as e.g., the convex potentials and the power-law or anharmonic potentials. Second, the dependence of the Rényi and Shannon uncertainty relations on the space and spin dimensionality effects; it seems that we could obtain it in our scenario provided the Rényi and Shannon uncertainty sums are expressed in terms of the Heisenberg uncertainty product, but this has not yet been found to the best of our knowledge. Third, the formulation of the quantum uncertainty principle by means of the position and momentum Fisher informations.

Finally, it would be interesting to extend a similar study to other classical entropy-like entropies (such as the Tsallis entropies and Salicrú-like extensions [175,176]) and their quantum-information generalizations (von Neumann, quantum Fisher, quantum Rényi, quantum Tsallis and extensions [177]) which play a prominent role in classical nonequilibrium thermodynamics [174] and modern quantum technologies (see e.g., [77,78]), respectively.

## Data Availability

Not applicable.
